# A meta-analysis indicates that the regulation of cell motility is a non-intrinsic function of chemoattractant receptors that is governed independently of directional sensing

**DOI:** 10.3389/fimmu.2022.1001086

**Published:** 2022-10-20

**Authors:** José Luis Rodríguez-Fernández, Olga Criado-García

**Affiliations:** Centro de Investigaciones Biológicas Margarita Salas, Consejo Superior de Investigaciones Científicas, Madrid, Spain

**Keywords:** chemoattractant, chemoattractant receptor, chemotaxis, chemoattraction, directional sensing, motility, migration, actin

## Abstract

Chemoattraction, defined as the migration of a cell toward a source of a chemical gradient, is controlled by chemoattractant receptors. Chemoattraction involves two basic activities, namely, directional sensing, a molecular mechanism that detects the direction of a source of chemoattractant, and actin-based motility, which allows the migration of a cell towards it. Current models assume first, that chemoattractant receptors govern both directional sensing and motility (most commonly inducing an increase in the migratory speed of the cells, i.e. chemokinesis), and, second, that the signaling pathways controlling both activities are intertwined. We performed a meta-analysis to reassess these two points. From this study emerge two main findings. First, although many chemoattractant receptors govern directional sensing, there are also receptors that do not regulate cell motility, suggesting that is the ability to control directional sensing, not motility, that best defines a chemoattractant receptor. Second, multiple experimental data suggest that receptor-controlled directional sensing and motility can be controlled independently. We hypothesize that this independence may be based on the existence of separated signalling modules that selectively govern directional sensing and motility in chemotactic cells. Together, the information gathered can be useful to update current models representing the signalling from chemoattractant receptors. The new models may facilitate the development of strategies for a more effective pharmacological modulation of chemoattractant receptor-controlled chemoattraction in health and disease.

“We shall not cease from exploration
And the end of all our exploring
Will be to arrive where we started
And know the place for the first time”
from “*Little Gidding*” by T.S. Elliot

## Introduction

Chemoattraction is defined as cell movement toward a gradient of increasing chemical concentration (1–4). Chemoattraction is controlled by specific receptors belonging largely to the G-protein coupled receptor (GPCR) and the Receptor Tyrosine kinase (RTK) families. The ability of chemoattractant receptors to govern chemoattraction “ensures that the right cells get to the right place at the right time” (3), explaining why this function plays such a key role in multiple biological processes. In many unicellular organisms, chemoattraction is required for foraging, in mammals is necessary for organ development during embryogenesis, for sperm migration toward the egg, for neurite outgrowth in the nervous system, for epithelial, and fibroblast cell migration during wound repair in the skin. In the immune system, it is crucial for the correct location of the leukocytes in different tissues during homeostasis and inflammation. Multiples pathologies, including metastatic colonization of cancer cells and inflammatory diseases, can be initiated or aggravated by abnormal stimulation of chemotaxis (5–8). Although eukaryotic chemotaxis was discovered in the second half of the XIX century by the German scientist Wilhelm Pfeffer (9, 10), the mechanisms controlling it have not been completely clarified.

Chemoattraction involves two activities, namely, directional sensing and motility. Directional sensing can be defined as a molecular mechanism whereby a cell detects the direction of a source of a ligand that in this context is called chemoattractant (11–16). Motility is an actin cytoskeleton-mediated process that allows the migration of a chemotactic cell in the direction pointed by the directional sensing machinery (17–20). Chemoattractant receptors control directional sensing in chemotactic cells, that is, they govern the molecular machinery that orients a cell toward a gradient of a chemoattractant (21–27). Chemoattractant receptors, in addition to directional sensing, can potentially control the motility of the cells. In this regard, it is well known that chemoattractant receptors can induce an increase in the migratory speed of the chemotactic cells, an effect called chemokinesis (28, 29). Interestingly, single-cell studies show that stimulation of chemoattractant receptors can also lead to inhibition of the speed of cells or to their repulsion away from a chemoattractant (30, 31). Hereafter, we will focus largely on chemokinesis, which is the effect on motility most commonly elicited by these receptors. Hence, hereafter when we indicate that a receptor controls cell motility, we refer to the induction of chemokinesis, unless otherwise it is stated.

Examination of established models describing the functions controlled by chemoattractant-receptors and the signaling pathways controlling these functions (15, 32–36), shows that it is largely accepted *first*, that chemoattractant receptors govern both directional sensing and actin-controlled motility (chemokinesis), and *second*, that the signaling pathways controlling both activities are intertwined (15, 32–36). We decided to perform a meta-analysis to reassess these two points. The results obtained question these two features of current models of chemoattractant receptors, and provide interesting information on the regulation of directional sensing and motility in chemotactic cells. Regarding the first point, it was found that although chemoattractant receptors largely control directional sensing, not all of them regulate chemokinesis, implying that these cells use their spontaneous motile machinery to migrate toward the chemoattractant. These results also indicate that the basic activity that defines chemoattractant receptors is their ability to control directional sensing. We suggest that the regulation of motility is an additional activity that could be controlled by these receptors in specific contexts. Hence, current models, which largely display chemoattractant receptors that control both directional sensing and chemokinesis, reflect only a functional subtype of chemoattractant receptors. Regarding the second point mentioned above, contrasting with current models, the data gathered suggest that directional sensing is regulated independently of basal or chemokinetic motility. We also present data suggesting that this independence could be based on the existence of separated signaling modules selectively governing directional sensing and motility. Together, the information gathered, that we describe in detail below, should be useful to update current models representing chemoattractant receptor-dependent signaling governing chemotaxis.

## Signaling molecules involved in the regulation of directional sensing in chemotactic cells

When a chemotactic cell detects a chemoattractant gradient, some signalling molecules selectively accumulate in the cell region exposed the first to this gradient. These molecular clusters seem to play a role in directional sensing because following their formation, the cell directs its movement toward the highest concentration of the chemoattractant. Hence, formation of these clusters of signalling molecules, which can be generated from precursors already present in the membrane or that could translocate from other cell regions, can be used as markers of the activation of the directional sensing molecular machinery in a chemotactic cell (37, 38). In this section we briefly introduce some of these molecular markers. The first candidates analyzed as possible directional sensing mediators were GPCRs and associated proteins. GPCRs can potentially couple with four families of heterotrimeric G proteins, namely G_s_, G_q_, G_12/13_, and G_i_ (39). G proteins include a α subunit and a βγ dimer, with the β and γ subunits bound tightly, but non-covalently. Considering that the inhibition of Gi proteins results in the blocking in most cases of the chemotaxis (24, 27), it was suggested that the GPCRs and Gi family members and, more specifically, the βγ subunits or, as indicated more recently, the α_i_ proteins, could behave as directional sensing mediators (24, 27, 40, 41). However, interestingly, in chemotactic cells exposed to gradients of chemoattractants, GPCRs and their associated G proteins seem to distribute evenly through the plasma membrane, suggesting that they are not key elements of the directional sensing molecular machinery (42–44). Although multiple signaling molecules have been suggested to be gradient sensors (15, 33, 45, 46), herein, we focus on the molecules that more often has been considered to play this role downstream of chemoattractant receptors, namely, the small guanosine triphosphatase (GTPase) Ras, and phosphatidylinositol (3–5)-trisphosphate (PIP3), a lipid product generated by class I phosphoinositide 3-kinases (PI3K).

Concerning Ras, it is possible to analyze the site of the cell membrane where this molecule is activated by transfecting cells with DNA constructs that encode the Ras-binding domain (RBD) of Raf1, a protein domain that selectively binds to active Ras (Ras-GTP), fused to a fluorescent protein. In cells expressing these constructs, it was observed that Ras became activated at the side of the cells first exposed to gradients of different types of chemoattractants (47–50). For instance, it has been observed that Ras can be a mediator of directional sensing in response to gradients of N-formyl-Met-Leu-Phe (fMLF) and adenosine 3′,5′-monophosphate (cAMP) in neutrophils and *Dictyostelium*, respectively (51, 52). To identify the sites at the plasma membrane where PIP3 is generated, researchers have transfected the chemotactic cells with constructs encoding pleckstrin homology (PH) domain, which displays a high affinity for PIP3, associated with a fluorophore protein. In studies carried out with *Dictyostelium*, neutrophils, or fibroblasts transfected with these constructs, it was observed that PIP3 localizes at the side of the cells first exposed to gradients of different chemoattractants (53–58). In this regard, for instance, it was shown that PI3K/PIP3 could mediate directional sensing in response to gradients of cAMP in *Dictyostelium*(53, 54, 56, 57), fMLF in neutrophil (55), and Platelet-derived growth factor (PDGF) in fibroblasts (58). Although initially it was considered that PI3K/PIP3 could be general regulators of directional sensing in chemotactic cells (53–63), subsequent studies have shown that GPCRs- or RTK-mediated directional sensing could still be observed even upon the complete pharmacological inhibition or the simultaneous knocking down of several PI3K isoforms (64–71). Hence, although PI3K/PIP3 could play an important role in the modulation of directional sensing in specific contexts, these molecules cannot be considered as universal regulators of directional sensing (64–70). In addition to the Ras family of proteins and the pair PI3K/PIP3, several other molecules that may also mediate directional sensing in *Dictyostelium* and other cell types have been described. Among these molecules are included the target of rapamycin complex 2 (TorC2), the Ras-Guanine nucleotide exchange factor (GEF) Aimless, the phospholipase A2 (PLA2), the soluble guanylyl cyclase (sGC) and its product cyclic guanosine monophosphate (cGMP) (45). In summary in cells and contexts in which Ras, PI3K/PIP3, and downstream PH-binding proteins, as well as other markers like TorC2, Aimless, PLA2, sGC or cGMP could govern directional sensing (45, 47, 54, 55), the accumulation of any of these molecules at the membranes sites first exposed to a gradient of chemoattractant could be used as proxy indicators of the activation of the directional sensing molecular machinery.

## Actin cytoskeleton regulatory components that govern cell motility

In this section we analyze briefly key molecular regulators of actin organization involved in the control of cell motility. Recently, single animal cell motility has been divided, based a variety of phenotypical characteristics, into two broad categories, namely, amoeboid and mesenchymal (72–74). Amoeboid motility (observed e.g. in *Dictyostelium*, mature dendritic cells (DCs), neutrophils, and cancer cells) is characterized by low contractility that is restricted mainly to the rear of the cell, weak adhesion to the substrate, lack of stress fibers, and high migratory speed ( 10 μm/min) (73). Mesenchymal motility (observed e.g. in fibroblasts and macrophages) is characterized by a high contractility based on the presence of actin stress fibers, strong adhesion to the substrate, actin-rich leading-edge structures, including lamellipodia and filopodia, and low migratory speed (< 1 μm/min) (73). Furthermore, cells in the organism may migrate either on two-dimensional (2D) flat surfaces, e.g. on lymphatic and blood vessels, or in three-dimensional (3D) environments, e.g. cells that migrate during embryonic development, metastatic processes, or during immune surveillance. Motility on flat surfaces has been extensively studied during the last decades (18, 75, 76). This type of motility generally involves the pushing forward of the cell membrane at the leading edge where protrusions called lamellipodia are formed. Adhesion receptors on lamellipodia attach to the extracellular matrix (ECM), allowing adhesion-mediated traction. At the same time that these processes take place at the cell´s front, at the cell´s rear retracts the trailing edge. The information available on 3D motility is still relatively sparse (77–79). However, it has been observed that unlike 2D migration, 3D motility can take place even in the absence of integrin-mediated adhesion and contractility (80–82). Moreover, compared with the cells migrating on 2D surfaces, which show at the leading edge largely lamellipodia and filopodia, the front of cells migrating in 3D environments displays a higher variety of protrusions, which include pseudopodia (77), cylinder-shaped structures called lobopodia (83), blebs (82), filopodia, and ruffle like structures (80). Furthermore, interestingly, in 3D environments cells can change the types of protrusion that they display as they migrate (77, 82).

**Figure 1:**
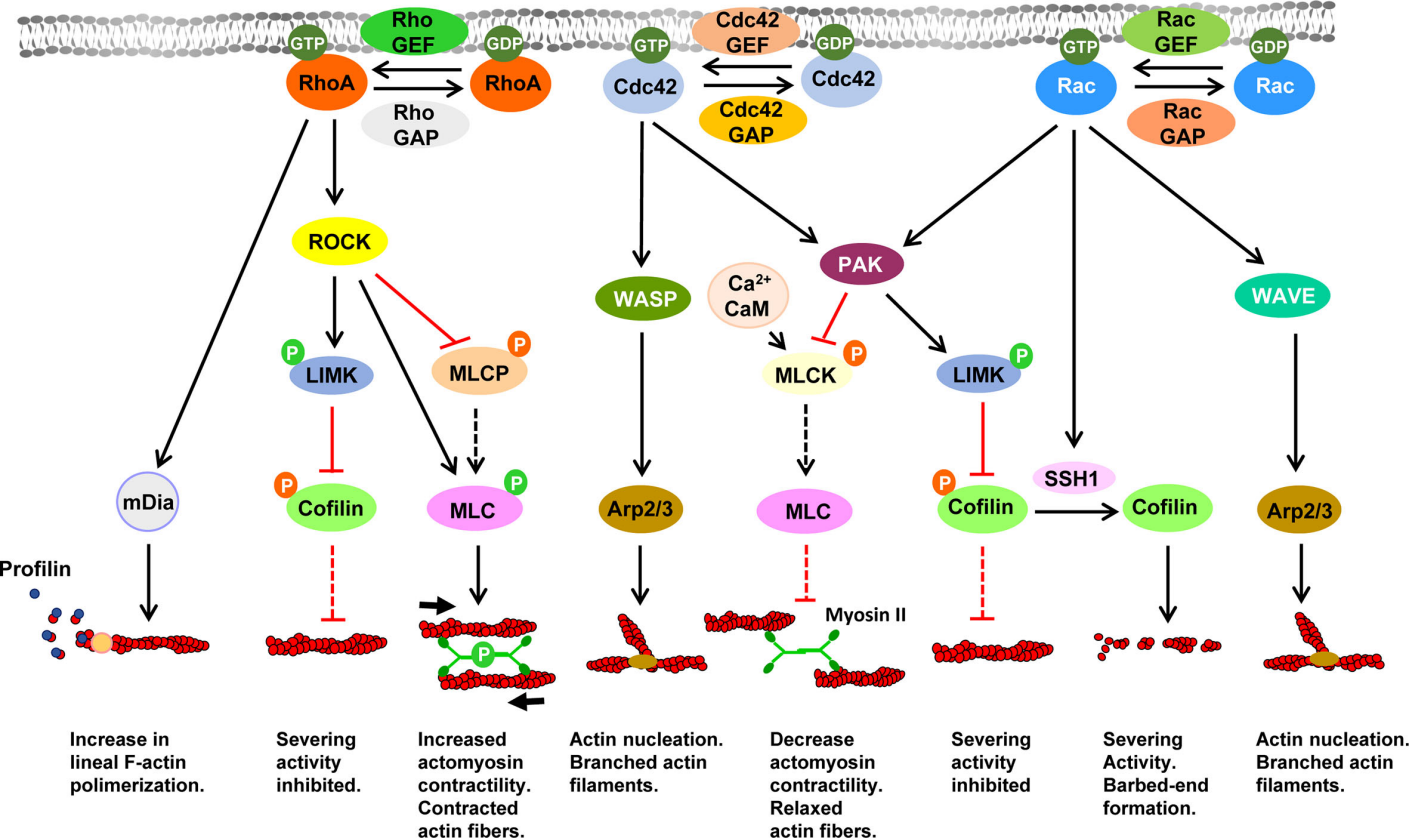
Signaling pathways controlling actin dynamics downstream of the Rho family of small GTPases Black and red lines indicate stimulatory and inhibitory effects, respectively. Dashed black or red lines indicate that upon inhibition of a specific molecule, ceases the stimulatory or inhibitory effect exerted by this molecule on its direct downstream target. In the lower part of the figure is indicated the effect of the indicated pathway on F-actin. Abbreviations: Arp2/3, Actin Related Protein 2/3 complex; CaM, Calmodulin; Cdc42, Cell Division Cycle 42; GAP, GTPase-activating proteins; GDP, Guanosine diphosphate; GEF, Guanine nucleotide exchange factors; GTP, Guanosine-5’-triphosphate; LIMK, LIM Motif-Containing Protein Kinase; mDia, mammalian Diaphanous-related formin; MLC, myosin light chain; MLCK, myosin light-chain kinase; MLCP, Myosin Light-Chain Phosphatase; PAK, p21-activated kinase; Rac, Ras-Related C3 Botulinum Toxin Substrate; RhoA, Ras homolog family member A; ROCK, Rho-associated protein kinase; SSH1, slingshot protein phosphatase 1; WASP, Wiskott-Aldrich Syndrome protein; WAVE, WASP-family verprolin-homologous protein.

Filamentous actin (F-actin), a key player in motility, is formed by units of globular actin (G-actin) that assemble to form a characteristic double helical filament (**Figure 1**). F-actin present two ends that display different characteristics, namely, a barbed or (+) end, where G-actin-GTP molecules are added, allowing filament growth, and a pointed or (–) end, where the F-actin shrinks. While the pointed end is generally located toward the cell interior, the barbed end is closer to the cell membrane, allowing that the growing filament may push the membrane forward (17, 84). Both pushing (compressive/protrusive) forces, mediated by actin polymerization, and pulling (tensile/contractile) forces, mediated by non-muscle MyoII acting on F-actin, are the main actin-mediated forces controlling motility (see below). Multiple actin regulatory molecules govern the dynamic changes in the actin cytoskeleton involved in cell motility (20). As indicated in **Figure 1**, three small GTPase families, namely, Cdc42, Rac and RhoA, activated near the plasma membrane, play a key role in the control of the different actin networks driving motility. These three GTPases are regulated by Guanine nucleotide exchange factors (GEFs) and GTPase-activating proteins (GAPs), which, respectively, yield GTP-bound (active), and GDP-bound (inactive) forms of these molecules. Downstream of Rac and Cdc42, the nucleation promoting factors (NPFs) Wiskott-Aldrich Syndrome protein (WASP), and WASP family verprolin homologous protein 1 or 2 (WAVE) induce activation of the actin nucleator Actin Related Protein 2/3 (Arp2/3) complex, which generates at the lamellipodia branched actin networks that push the cell membrane forward (85–88). Downstream of RhoA another actin nucleator mammalian Diaphanous Related Formin (mDia), promotes actin nucleation and accelerates the elongation of actin filaments by transferring the G-actin monomers bound to profilin to F-actin barbed ends (89–92). Downstream of Cdc42 and Rac, the serine-kinase p21-activated kinase (PAK) (93, 94) and downstream of RhoA, the kinase Rho-associated protein kinase (ROCK) (95), mediate the activation of the LIM-domain kinases (LIMKs). LIMKs induce phosphorylation/inactivation of the actin severing protein cofilin, preventing it to exert severing effects on F-actin. Moreover, Rac-dependent activation of slingshot1 (SSH1) by catalyzing cofilin dephosphorylation opposes the inhibitory effects of LIMK, leading to an increase in cofilin actin severing activity and actin depolymerization (96). PAK also induces inactivation of the Ca^2+^/calmodulin (CaM)-dependent myosin light chain kinase (MLCK), which promotes phosphorylation/activation of the myosin light chain (MLC) (97, 98) that drives myosin II (Myo II)-dependent contractility. The kinase ROCK also induces phosphorylation and inactivation of the Myosin Light-Chain Phosphatase (MLCP) regulatory subunit (MYPT1), which prevents that this phosphatase may dephosphorylate and inactivate MLC (99). Actomyosin-dependent contractility generates the tension necessary for the retraction of the cell´s rear-end, the maintenance of membrane tension and cell shape (76). Finally, emphasizing the key importance of actin organization and its molecular regulators, 2D motility is inhibited upon disruption of F-actin organization with pharmacological inhibitors (100, 101) or upon perturbation of some of molecular regulators of actin, including Rac1 and Rac2 (71, 102), dedicator of cytokinesis (DOCK2), a GEF for Rac (103, 104), Cdc42 (71), RhoA (65), GEF-H1, a GEF for RhoA/Rac (105), ROCK (51, 101), MyoII (68, 101), mDia (106), WASP (107, 108), the Arp2/3 complex (88), cofilin (105), Slinshot (105), and profilin (105). Interestingly, in cells that migrate in 3D environments, inhibition of some of aforementioned actin regulators, including RhoA (109), ROCK (80, 100, 109, 110), MyoII (80, 110), mDia1 (111), WAVE (112), also lead to impairment of the motility. These results point out a set of actin regulatory molecules that selectively control both 2D and 3- motility (see below).

## The regulation of motility is not an obligatory function of chemoattractant receptors

We asked whether as commonly assumed in most available models describing the signalling pathways regulating chemotaxis, the ability to increase the migratory speed of the cells (chemokinesis) is an intrinsic activity of chemoattractant receptors. For this purpose, we carried out a meta-analysis in which we studied the ability of transfected or endogenous expressed chemoattractant receptors to govern this activity. The results obtained in multiple settings using a variety of experimental strategies (**Tables 1**, **2**), including methods that allow a direct visualization and measurement of the migratory speed of the cells (**Table 2**), suggests that the ability to induce chemokinesis is not an intrinsic activity of chemoattractant receptors (30, 121, 132–136, 138, 139).

**Table 1:** Ability of transfected chemoattractant receptors to control chemokinesis.

Transfected chemo-attractant receptor	Chemo-attractant	Cell model	Chemoattractant receptor governs:		Method used to measure:		Refs
			Directional sensing	Motility (chemokinesis)	Directional sensing	Migratory speed
CCR4	CCL17	HEK-293	YES	YES	Microchemotaxis chamber assays	Microchemotaxis chamber-based checkerboard assays	(22)
CXCR1	CXCL8	BCs (L1/2 cells)	YES	YES	Transwell assays	Transwell assay-based checkerboard analyzes	(21)
CXCR2	CXCL8	HEK-293	YES	YES	Microchemotaxis chamber assays	Microchemotaxis chamber-based checkerboard assays	(26)
C5aR	C5a	BCs (L1/2 cells)	YES	YES	Transwell assays	Transwell assay-based checkerboard analyzes	(21)
CCR1	CCL3	BCs (L1/2 cells)	YES	NO	Transwell assays	Transwell assay-based checkerboard analyzes	(21)
CCR2	CCL2	BCs (300-19 cells)	YES	NO	Transwell assays	Transwell assay-based checkerboard analyzes	(23, 24)
CCR8	CCL8	BCs (4DE4 cells)	YES	NO	Microchemotaxis chamber assays	Microchemotaxis chamber-based checkerboard assays	(25)
CXCR1	CXCL8	TCs (Jurkat cells)	YES	NO	Transwell assays	Transwell assay-based checkerboard analyzes	(21)
fMLF-R	fMLF	BCs (L1/2 cells)	YES	NO	Transwell assays	Transwell assay-based checkerboard analyzes	(21)
D2 dopamine receptor	Quinpirole	HEK-293	YES	NO	Boyden chambers	Boyden Chamber-based checkerboard analyzes	(27)
δ Opioid receptor	DADLE	HEK-293	YES	NO	Boyden chambers	Boyden Chamber-based checkerboard analyzes	(27)
µ Opioid receptor	Etorphine or morphine	HEK-293	YES	NO	Boyden chambers	Boyden Chamber-based checkerboard analyzes	(27)
κ Opioid receptor	U-50488	BCs (300-19 cells)	YES	NO	Transwell assays	Transwell assay-based checkerboard analyzes	(24)

Transfected chemoattractant receptors that control directional sensing and chemokinesis (clear grey boxes) or directional sensing, but not chemokinesis (dark grey boxes). BC, B cells; C5aR, C5a receptor complement component 5a receptor 1; DADLE, [D-Ala (2)-D-Leu (5)]-enkephalin; fMLF, N-formyl-methionyl-leucyl-phenylalanine; fMLF-R, fMLF receptor; HEK-293, human embryo kidney cells; L1/2 cells, murine pre-BCs; 4DE4 cells, mouse pre-BCs; TC, T cells; 300-19 cells, murine pre-BCs.

**Table 2:** Ability of endogenous chemoattractant receptors to control chemokinesis In the table are presented endogenous chemoattractant receptors that control directional sensing and chemokinesis (clear grey) or directional sensing, but not chemokinesis (dark grey).

Endogenous chemoattractant receptor	Chemo-attractant	Cell model	Chemoattractant receptor governs:		Method used to measure:		Refs
			Directional sensing	Motility (chemokinesis)	Directional sensing	Migratory speed	
CCR2	CCL2	hNK (3.3 cells)	YES	YES	Microchemotaxis chamber assays	Microchemotaxis chamber-based checkerboard assays	(113)
CCR1/CCR3/ CCR5	CCL5	hNK (3.3 cells)	YES	YES	Microchemotaxis chamber assays	Microchemotaxis chamber-based checkerboard assays	(113)
CCR1/ CCR5	CCL3	mNeu	YES	YES	Transwell Assays	Transwell assay-based checkerboard analyzes	(114)
CCR1/ CCR5	CCL3	hNeu	YES	YES	Time-lapse microscopy in Zigmond Chambers	Time-lapse microscopy in Zigmond Chambers	(115)
CCR7	CCL19/ CCL21	mTCs	YES	YES	Intravital 2-photon laser microscopy	Intravital 2-photon laser microscopy	(116)
CCR7	CCL21	mTCs	YES	YES	Transwell assays	Transwell assay-based checkerboard analyzes	(117)
CCR7	CCL19/ CCL21	hDCs (Mono-DCs)	YES	YES	Transwell assays	-Time lapse microscopy -Transwell assay-based checkerboard analyzes	(65)
CCR7	CCL21	mDCs (BM-DCs)	YES	YES	Transwell assays	Transwell assay-based checkerboard analyzes	(117)
CXCR1/CXCR2	CXCL8	hNeu	YES	YES	Time-lapse microscopy in 3D collagen gels	Time-lapse microscopy in 3D collagen gels	(118)
CXCR1/CXCR2	CXCL8	hNeu	YES	YES	Boyden chambers	Boyden Chamber-based checkerboard analyzes	(119)
CXCR1/CXCR2	CXCL8	hNeu	YES	YES	Time-lapse microscopy in 3D μ-Slide migration chambers	Time-lapse microscopy in 3D μ-Slide migration chambers	(120)
CXCR3	CXCL10	hTCs	YES	YES ^(1)^	Time-lapse microscopy in microfluidic devices	Time-lapse microscopy in microfluidic devices	(121)
fMLF-R	fMLF	mNeu	YES	YES	Time-lapse microscopy in Zigmond Chambers	Time-lapse microscopy in Zigmond chambers	(102)
fMLF-R	fMLF	mNeu	YES	YES	Transwell Assays	Transwell assay-based checkerboard analyzes	(114)
fMLF-R	fMLF	hNeu	YES	YES	Time-lapse microscopy in Zigmond Chambers	Time-lapse microscopy in Zigmond chambers	(115)
fMLF-R	fMLF	hNeu	YES	YES	Time-lapse microscopy in 3D μ-Slide migration chambers	Time-lapse microscopy in 3D μ-Slide migration chambers	(120)
cAR1	cAMP	Dicty	YES	YES	Time-lapse microscopy (home-made chambers)	Time-lapse microscopy (home-made chambers)	(122)
cAR1	cAMP	Dicty	YES	YES	Video microscopy using Sykes-Moore chambers	Video microscopy using Sykes-Moore chambers	(123)
cAR1	cAMP	Dicty	YES	YES	Time-lapse microscopy in Zigmond Chambers	Time-lapse microscopy in Zigmond chambers	(124)
fAR1	Folic acid	Dicty	YES	YES	Time-lapse microscopy in cellophane square test EZ-TAXIScans	Time-lapse microscopy in Cellophane square test EZ-TAXIScans	(125–128)
C5aR	C5a	hNeu	YES	YES ^(2)^	Time-lapse microscopy in microfluidic devices	Time-lapse microscopy in microfluidic devices	(30)
CCR1/CCR3/ CCR5	CCL5	mMΦ (BM-MΦ)	YES	NO	Electrical cell impedance-based assays	Electrical cell impedance-based checkerboard analyzes	(129)
CCR1/CCR3/ CCR5	CCL5	hNK (IANK cells)	YES	NO	Microchemotaxis chamber assays	Microchemotaxis chamber-based checkerboard assays	(113)
CCR1/CCR5	CCL3	hNK (3.3 cells)	YES	NO	Microchemotaxis chamber assays	Microchemotaxis chamber-based checkerboard assays	(113)
CCR1/CCR5	CCL3	hNK (IANK cells)	YES	NO	Microchemotaxis chamber assays	Microchemotaxis chamber-based checkerboard assays	(113)
CCR2	CCL2	hNK (IANK cells)	YES	NO	Microchemotaxis chamber assays	Microchemotaxis chamber-based checkerboard assays	(113)
CCR4	CCL17	hTCs (Hut78)	YES	NO	Microchemotaxis chamber assays	Microchemotaxis chamber-based checkerboard assays	(130)
CCR5	CCL4	hMΦ (mono-MΦ)	YES	NO	Transwell assays	Transwell assay-based checkerboard analyzes	(131)
CXCR3	CXCL10	hTCs	YES	NO ^(3)^	Time-lapse microscopy in microfluidic devices	Time-lapse microscopy in microfluidic devices	(121)
CXCR4	CXCL12	hDCs (mono-DCs)	YES	NO	Transwell assays	Time-lapse microscopy Transwell assay-based checkerboard analyzes	(132)
CXCR4	CXCL12	mDCs (BM-DCs)	YES	NO	Transwell assays	Transwell assay-based checkerboard analyzes	(117)
CXCR4	CXCL12	mBCs	YES	NO	Time-lapse microscopy in 3D collagen gels	Time-lapse microscopy in 3D collagen gels	(133)
CXCR4	CXCL12	hHSPCs (CD133+ hUCB-HSPCs)	YES	NO	Time-lapse microscopy in 3D collagen gels	Time-lapse microscopy in 3D collagen gels	(134)
C5aR	C5a	hNeu	YES	NO ^(4)^	Time-lapse microscopy in microfluidic devices	Time-lapse microscopy in microfluidic devices	(30)
VEGF-R	VEGF	mMCs (BM-MCs)	YES	NO	Transwell assays	Time lapse microscopy Transwell assay-based checkerboard analyzes	(135)
VEGF-R2	VEGF	rNeural progenitors (FGF2-stimulated SVZ cells)	YES	NO	Time-lapse microscopy in Dunn chambers	Time-lapse microscopy in Dunn chambers	(136)
PDGF-R	PDGF	hNeural precursor cells	YES	NO	Transwell assays	Chemokinetics tracks	(137)

See also legend to **Table 1**. Notes ^(1)^: Chemokinesis was observed only when the T cells are exposed to a concentration gradient of CXCL10 (see text for details) ^(2)^. Chemokinesis was observed when the cells were exposed to a low concentration gradient (1 nM) of C5a. (3) Chemokinesis was not observed when instead of a gradient of CXCL10, the T cells were immersed in a medium with this chemoattractant. ^(4)^ Chemokinesis was not observed when the cells are exposed to a high concentration gradient (100 nM) of C5a. BM, Bone marrow; BM-DCs, Bone marrow-derived murine dendritic cells; BM-MCs, murine BM-derived mononuclear cells; BM-MΦ, bone-marrow-derived murine macrophages; cAMP, Cyclic adenosine monophosphate; cAR1, cyclic AMP receptor 1; DCs, dendritic cells; Dicty, *Dictyostelium discoideum*; fAR1, folic acid receptor 1; hDCs; human DCs; FGF2, Fibroblast growth factor-2; hMΦ, human macrophages; hNeu, human neutrophils; Hut78, human T cells; HSPCs, hematopoietic stem/progenitor cells; hUCB-HSPCs, human umbilical cord blood CD133+ HSPCs; hTCs, human TCs; hNK, human natural killer cells; IANK, human IL-2-activated NK cells; mBCs, murine BCs; mBM, murine BM; mMCs, murine mononuclear cells; mDCs, murine DCs; mMΦ, murine macrophages; mNeu, murine neutrophils; Mono, monocytes; mono-DCs, human monocyte-derived dendritic cells; mono-MΦ, human monocyte-derived MΦ; mTCs, murine TCs; pMΦ, peritoneal macrophages; PDGF, platelet-derived growth factor; PDGF-R; platelet-derived growth factor receptor; TC, T cells; VEGF, vascular endothelial growth factor; VEGF-R, vascular endothelial growth factor receptor; SVZ cells, rat subventricular zone cells; 3.3 cells, human NK cell line.

**Figure 2:**
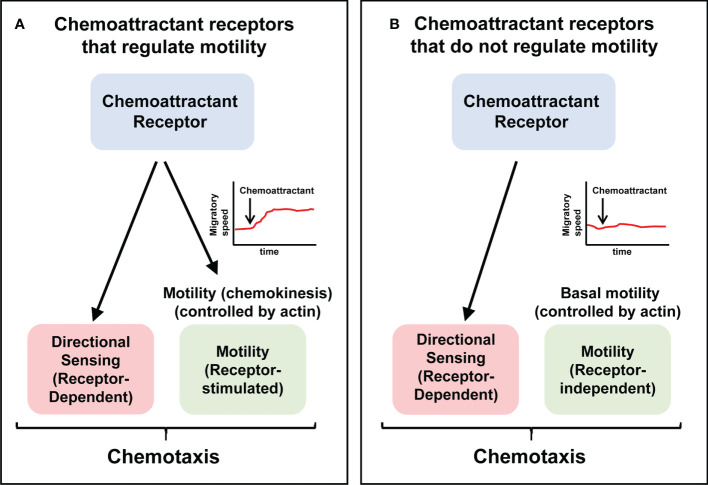
The ability to control chemokinesis is not an obligatory activity of chemoattractant receptors Chemotaxis involves directional sensing and motility. Chemoattractant receptors can be functionally classified into two groups. **(A)** Receptors that control both directional sensing and motility (chemokinesis). **(B)** Receptors that control directional sensing, but not chemokinesis, implying that stimulation of these receptors do not alter the speed of the cells. Current models of chemoattractant receptors-mediated signaling are largely based on the receptors shown in **(A)** See **Tables 1**, **2**, for examples of both types of receptors.

The transfection of cells with a chemoattractant receptor not expressed in them induce their directed migration towards specific ligands of this receptor/s (21–27). In these experiments it could be observed that the transfected chemoattractant receptors could be functionally classified into two subgroups (**Table 1** **)**. A first subgroup includes receptors that govern both directional sensing and motility (chemokinesis) (**Figure 2**). A second subgroup include receptors that regulate directional sensing, but not chemokinesis, suggesting that they use their basal spontaneous motility to migrate towards the source of a chemoattractant (**Figure 2**).

The following transfected chemoattractant receptors belong to the first subgroup (**Figure 2**; **Table 1**). CXCR1-transfected and C5aR-transfected mouse leukaemia L1/2 BCs in response to CXCL8 and C5a, respectively (21); CCR4-transfected and CXCR2-transfected HEK-293 cells in response to CCL17 and CXCL8, respectively (22, 26) (**Table 1**). The following transfected chemoattractant receptors belong to the second subgroup (**Figure 2**; **Table 1**). CCR1-transfected leukaemia L1/2 BCs, in response to CCL3 (21); CCR2-transfected in 300-19 BCs, in response to CCL2 (23, 24); CCR8-transfected 4DE4 BCs, in response to CCL8 (25); CXCR1-tranfected Jurkat T cells, in response to CXCL8 (21); fMLF-R-transfected leukaemia L1/2 BCs in response to fMLF (21); HEK-293 cells transfected either with the D2 dopamine receptor (in response to the ligand quinpirole); or the δ-opiod receptor (in response to the ligand DADLE), or the μ-opiod receptor (in response to the ligands etorphine or morphine) (27); and finally 300-19 BCs transfected with the κ-opiod receptor (in response to the ligand U-50488) (24).

As in the case of the transfected receptors, endogenously expressed chemoattractant receptors can be also classified into the two functional groups mentioned above (**Table 2**). The following endogenous receptors belong to the subgroup that control both directional sensing and chemokinesis in response to their ligands (presented in brackets) (**Figure 2**; **Table 2**): CCR2 (ligand CCL2) in Natural Killer (NK) cells (113); CCR1/CCR3/CCR5 (ligand CCL5) in NK Cells (113); CCR1/CCR5 (ligand CCL3) in neutrophils (114, 115); CCR7 (ligands CCL19, CCL21) in T cells (116, 117) and DCs (65, 117); CXCR1/CXCR2 (ligand CXCL8) in neutrophils (118–120); CXCR3 (ligand CXCL10), in T cells when CXCL10 is presented to these cells in the form of a concentration gradient (121) (see below, see also **Table 2** and legend); C5a-R (ligand C5a), in neutrophil when C5a is presented to the cells at a low concentration gradient (1 nM) (see below the effect of exposing cells at a high concentration of C5a) (30), the fMLF-R (ligand fMLF) in neutrophils (102, 114, 115, 120); and the cAMP receptor (cAR1) (ligand cAMP) in *Dictyostelium*(122–124).

Regarding the endogenously expressed chemoattractant receptor that regulate directional sensing, but not chemokinesis (**Figure 2**), interestingly, one of the earliest examples date back to the year 1936 (138). In this study, the authors used “cinemicrography” to analyze the chemoattractive behavior of individual human polymorphonuclear (PMN) leucocytes in response to *Staphylococcus albus*. Although unknown to the authors at the time, today we know that *Staphylococcus* bacteria released the chemoattractant fMLF, which attracts the PMN leucocytes. The authors point out: “The rate of locomotion is not affected by chemotaxis; thus, leukocytes move no more rapidly when responding to chemotactic stimuli than when moving at random. Thus chemotaxis does not affect the rate of amoeboid motion; rather is chemotaxis a directional response superimposed on amoeboid motion” (138). These earlier results were confirmed in another article, published in 1972 in which the authors stated: “The findings that the average speed of cells in the presence and absence of attractant did not differ, and that individual cells did not alter speed on approaching attractant, confirm the view of Dixon & McCutcheon that chemotaxis is a function only of direction of movement” (139). Additional examples of endogenously chemoattractant receptor that regulate directional sensing, but not chemokinesis, have been reported during the last years (see **Figure 2**, and **Table 2**). These examples include: CCR1/CCR3/CCR5 (ligand CCL5) in bone-marrow-derived macrophages (BM- MΦ) (129), CCR1/CCR5 (ligand CCL3) in NK cells and IL-2-stimulated NK cells (113), CCR2 (ligand CCL2) in IL-2-stimulated NK cells (113), CCR4 (ligand CCL17) in HUT-78 TCs (130), CCR5 (ligand CCL4) in human monocyte-derived MΦ (mono-MΦ) (131), CXCR3 (ligand CXCL10) in human T cells in experiments in which CXCL10 is presented uniformly dissolved in a solution and not as a gradient to these cells (**Table 2** and legend) (121); CXCR4 (ligand CXCL12), in human monocyte-derived DCs (mono-DCs), in murine bone-marrow-derived DCs, in murine BCs and in hematopoietic stem/progenitor cells (HSPCs) (117, 132–134), C5a-R (ligand C5a) when C5a is presented to the neutrophils at a high concentration gradient (100 nM) (30), the VEGF-R (ligand VEFG) in murine bone marrow-derived mononuclear cells (mBM-MC) (135) and in Fibroblast growth factor 2 (FGF2)-stimulated rat neural precursor cells (136), in PDGF-receptor (PDGF-R)-stimulated human neural precursor cells (137).

## The ability of a chemoattractant receptor to govern chemokinesis is context-dependent

The results presented above indicate that not all chemoattractant receptors regulate chemokinesis (**Tables 1**, **2**). Interestingly, experimental data presented in **Tables 1**, **2** suggest that the ability of chemoattractant receptor to govern chemokinesis could be context-dependent. For instance, the stimulation of CXCR1-transfected L1/2 BCs with CXCL8 induces both a directional response toward this ligand and chemokinesis, however, in CXCR1-transfected Jurkat T stimulated with a gradient of CXCL8 it is observed a directional response of the cells, but not chemokinesis (21) (**Table 1**). In fMLF-R-transfected L1/2 BCs, a gradient of fMLF promotes directional migration, but no chemokinesis (21) (**Table 1**), however, in neutrophils expressing endogenously the fMLF-R, the stimulation with fMLF promotes both directional migration and chemokinesis (114, 115, 120) (**Table 2**). Stimulation of CCR2 in NK cells (3.3 cells) with its ligand CCL2 induces a directional response toward this ligand and chemokinesis (113), however, the stimulation of CCR2 in IL-2-activated NK cells (IANK cells) with a gradient of CCL2 still induces a directional response towards this ligand, but not chemokinesis (113). The stimulation of the C5a-R (ligand C5a) in human neutrophils induces migration in the direction of C5a and also chemokinesis, but only at a low concentration gradient of this ligand (1 nM). However, when the neutrophils are exposed to a high concentration gradient of C5a (100 nM), they respond chemotactically, indicating that their directional sensing is not altered, however, the cells do not display a chemokinetic response (30) (**Table 2**). Likewise, when human neutrophils are exposed at different concentration gradients (12, 120, or 1200 nM) of CXCL8 (receptors CXCR1 and CXCR2), it was observed that the cells displayed a chemotactic response at all the aforementioned concentrations, and also chemokinetic responses at 120 or 1200 nM, but not at 12 nM concentration (30) (**Table 2**). Interestingly, even the way in which the chemoattractant is presented to the cells, either in the form of a gradient or homogeneously in media, may determine whether chemokinesis is induced. For instance, CXCL10 (receptor CXCR3) in addition to chemoattraction, also induces a chemokinesis both in resting and mitogen-activated human T cells when this chemokine is presented to the cells in the form of a gradient (**Table 2**), but not when these cells are bathed in a medium that contains CXCL10 (121) (**Table 2**). Interestingly, CXCR4 seems to fail to control chemokinesis in different cell types (117, 132–134) (**Table 2**). Further suggesting that CXCR4 does not govern chemokinesis, the blocking of this receptor in human HSPCs with the potent CXCR4 antagonist AMD3100, abrogated directional sensing, but did not affect the motility of the cells (134) (see also below and **Table 3**). In summary, the cell type, the activation or differentiation state of the cells, the concentration of the gradient of chemoattractant, or even the way in which this chemoattractant is presented to the cells, either as gradient or homogeneously in solution, may determine if a specific receptor induces chemokinesis.

**Table 3:** Independent regulation of directional sensing and motility downstream of the G-protein-coupled receptor family of chemoattractant receptors.

Target protein	Role of target protein	Tool used to block the target/s	Chemo-attractant	Chemo-attractant receptor(GPCRs)	Cellmodel	Effect of inhibition of the target on:		Method/device used to analyze:		Refs
						Directional sensing	Motility	Directional sensing	Motility	
CXCR4	Chemokine receptor	Inhibitor: AMD3100	CXCL12	CXCR4	HSPCs (CD133+ hUCB-HSPCs)	Inhibited	No Effect	Time-lapse microscopy in 3D collagen gels (using µ-Ibidis chambers)	Time-lapse microscopy in 3D collagen gels (using µ-Ibidis chambers)	(134)
RasG	GTPase, MAPK kinase pathway regulator	Knock-Out: RasG-/-	cAMP	cAR1	Dicty (AX2 cells)	Inhibited	No Effect	Monitorization of translocation of GFP-RBD to the PM by microscopy (cAMP delivered with a micropipette)	Time-lapse microscopy	(47)
PI3K, PLA2	Phosphoinositide-3-kinase, Phospholipase A2	Inhibitors: LY BPB	cAMP	cAR1	GC-/- Dicty (AX2 cells)	Inhibited	No Effect	Time-lapse microscopy in Zigmond chambers	Time-lapse microscopy in Zigmond chambers	(45)
PI3Kγ	Phosphoinositide-3-kinase	Knock-Out: PI3Kγ -/-	fMLF	FPR1	BM-Neu (mNeu)	No Effect	Inhibited	Time-lapse microscopy in TAXIScan assays	Time-lapse microscopy in TAXIScan assays	(140)
SHIP1	Inositol-5-phospatase	Knock-Out: ship1γ -/-	fMLF	FPR1	pMϕ (BM-Mϕs)	No Effect	Inhibited	Time-lapse microscopy in TAXIScan assays	Time-lapse microscopy in TAXIScan assays	(140)
Ras GEF (Aimless)	GEF for Ras	Knock-Out: Ras-GEF-/-	cAMP	cAR1	Dicty (AX3 cells)	Inhibited	No Effect	Konijn method	Video microscopy	(141)
Rac1	GTPase, actin regulator	Knock-Out: Rac1-/-	fMLF	FPR1 (1)	Neu (mNeu)	Inhibited	No Effect	Time-lapse microscopy in Zigmond chamber	Time-lapse microscopy in Zigmond chambers	(102)
Rac2	GTPase, actin regulator	Knock-Out: Rac2-/-	fMLF	FPR1	Neu (mNeu)	No effect	Inhibited	Time-lapse microscopy in Zigmond chambers	Time-lapse microscopy in Zigmond chambers	(102)
Cdc42	GTPase, actin regulator	Knock-Out: Cdc42-/-	CCL19	CCR7	DCs (BM-DCs)	No effect	Inhibited	Under agarose cell migration assays and 3D collagen gels	Time-lapse microscopy in under agarose cell migration assays and 3D collagen gels	(142)
RIC8	GEF for Gα	Knock-Out: Ric8-/-	cAMP	cAR1	Dicty (AX3 cells)	No effect	Inhibited	Time-lapse microscopy and micropipette cAMP delivery	Time lapse microscopy	(143)
F-Actin	Cytoskeletal component	Inhibitor: Cytoch. D	C5a	C5a-R	Mϕ (BM-Mϕs)	No Effect	Inhibited	Time-lapse microscopy in 3D collagen gels (using µ-Ibidis chambers	Time-lapse microscopy in 3D collagen gels (using µ-Ibidis chambers)	(100)
		Inhibitor: Latrun. A	CCL19	CCR7	DC (BM-DCs)	No effect	Inhibited	Time-lapse microscopy in microfluidic devices	Time-lapse microscopy in microfluidic devices	(101)
			cAMP	cAR1	Dicty	No effect	Inhibited	Monitorization of translocation of GFP-RBD to the PM by microscopy (cAMP delivered with a micropipette)	Time-lapse microscopy	(47)
			cAMP	cAR1	Dicty (AX3 cells)	No effect	Inhibited	Monitorization of translocation of PH-Crac-GFP or PI3K2-GFP to the PM by microscopy (cAMP delivered with micropipette)	Time-lapse microscopy	(59)
		Inhibitor: Latrun. A or Cytoch. D	cAMP	cAR1	Dicty (AX2 cells)	No effect	Inhibited	Monitorization of translocation of PH-Crac-GFP to the PM by microscopy (cAMP delivered with a micropipette)	Time-lapse microscopy	(44, 56, 144)
		Inhibitor: Latrun. B	fMLF	FPR1	Neu (HL-60)	No effect	No measured	Monitorization of translocation of PH-AKT-GFP to the PM by microscopy (fMLF delivered with a micropipette)	Time-lapse microscopy	(43)
RhoA	GTPase, actin regulator	Inhibitor: C3-exoenzyme	CCL19 or CCL21	CCR7	DC (Mono-DCs)	No Effect	Inhibited	Transwell assays	Time-lapse microscopy	(65)
		Inhibitor: TAT-C3	fMLF	FPR1	Mono (hMono)	No Effect	Inhibited	Time-lapse microscopy in 3D collagen gels (using µ-Ibidis chambers	Time-lapse microscopy in 3D collagen gels (using µ-Ibidis chambers)	(109)
GEF-H1 (ARHGEF2)	GEF for RhoA/Rac	siRNA: GEF-H1	fMLF	FPR1	Myeloid Leukemia (PLB-985)	No Effect	Inhibited	Time-lapse microscopy using under agarose cell migration assays	Time-lapse microscopy using under agarose cell migration assays	(105)
ROCK	Kinase, actin cytoskeleton regulator	Inhibitor: Y27632	CCL19	CCR7	DC (BM-DCs)	No Effect	Inhibited	Time-lapse microscopy in microfluidic devices	Time-lapse microscopy in microfluidic devices	(101)
					DC (BM-DCs)	No effect	Inhibited	Time-lapse microscopy in 3D collagen gels	Time-lapse microscopy in 3D collagen gels	(80)
					GRAN (mGRAN)					
					BC (mBCs)					
			CCL21	CCR7	DC (BM-DCs)	No effect	Inhibited	Time-lapse microscopy in 3D collagen gels	Time-lapse microscopy in 3D collagen gels and microchannels	(110)
			fMLF	FPR1	Mono (hMono)	No Effect	Inhibited	Time-lapse microscopy in 3D collagen gels (using µ-Ibidis chambers)	Time-lapse microscopy in 3D collagen gels (using µ-Ibidis chambers	(109)
			C5a	C5aR	Mϕ (BM-Mϕs)	No Effect	Inhibited	Time-lapse microscopy in 3D collagen gels (using µ-Ibidis chambers	Time-lapse microscopy in 3D collagen gels (using µ-Ibidis chambers	(100)
F-Actin ROCK	Cytoskeletal component/Kinase, actin regulator	Inhibitors: JLY (Jasplak., Latrun. B, Y27632)	fMLF	FPR1	Neu (HL-60)	No Effect	Inhibited	Monitorization of translocation of PH-AKT-GFP to the PM by microscopy (fMLF delivered with a micropipete)	Time-lapse microscopy	(51)
Myosin II	Actin-associated protein, controls contractility	Inhibitor: Blebbistatin	CCL19	CCR7	DC (BM-DCs)	No effect	Inhibited	Time-lapse microscopy in microfluidic devices	Time-lapse microscopy in microfluidic devices	(101)
		Inhibitor: Blebbistatin	CCL19	CCR7	DC (BM-DCs)	No Effect	Inhibited	Time-lapse microscopy in 3D collagen gels	Time-lapse microscopy in 3D collagen gels	(80)
					BC (mBCs)					
					GRANs (mGRANs)					
		Inhibitor: Blebbistatin	CCL21	CCR7	DC (BM-DCs)	No effect	Inhibited	Time-lapse microscopy in 3D collagen gels	Time-lapse microscopy in 3D collagen gels and microchannel analyzes	(110)
		Knock-Out: MyoII-/-								
mDia1	Actin polymerization regulator	Knock-Out: mDia1-/-	CCL21	CCR7	DC (BM-DCs)	No effect	Inhibited	Transwell assays	Time-lapse microscopy in TAXIScan assays	(106)
			CCL21	CCR7	DC (BM-DCs)	No effect	Inhibited	Time-lapse microscopy in 3D collagen gels	Time-lapse microscopy in 3D collagen gels	(111)
WASP	Actin nucleation promoting factor	shRNA: WASP	fMLF	FPR1	Neu (HL-60)	No Effect	Inhibited	Time-lapse microscopy in TAXIScan assays	Time-lapse microscopy in TAXIScan Assays	(107)
WAVE	Actin nucleation promoting factor	Knock-Out: Hem-/-	CCL19	CCR7	DC (BM-DCs)	No Effect	Inhibited	Time-lapse microscopy in 3D collagen gels	Time-lapse microscopy in 3D collagen gel	(112)
DOCK2	GEF for the cytoskeletal regulator Rac	Knock-Out: DOCK2-/-	fMLF	FPR1	Neu (mNeu)	No effect	Inhibited	Monitorization of translocation of PH-AKT-GFP to the PM by time-lapse microscopy, Zigmond chamber, Transwell assays	Time-lapse microscopy in Zigmond chambers	(103)
			C5a	C5aR	Neu (mNeu)	No effect	Inhibited	Monitorization of translocation of PH-AKT-GFP to the PM by time lapse microscopy, Transwell assays	Time-lapse microscopy in Zigmond chambers	(103)
			S1P	S1P-R	TC (mTCs)	No effect	Inhibited	Transwell assays	Time-lapse microscopy	(104)
Cofilin	Cytoskeletal regulator	siRNA: Cofilin	fMLF	FPR1	Myeloid leukemia (PLB-985)	No effect	Inhibited	Time-lapse microscopy in under agarose cell migration assays	Time-lapse microscopy using under agarose cell migration assays	(105)
Slingshot	Phosphatase, activates severing activity of cofilin	siRNA: Slingshot	fMLF	FPR1	Myeloid leukemia (PLB-985)	No effect	Inhibited	Time-lapse microscopy in under agarose cell migration assays	Time-lapse microscopy in under agarose cell migration assays	(105)
Profilin	Cytoskeletal regulator	siRNA: Profilin	fMLF	FPR1	Myeloid leukemia (PLB-985)	No effect	Inhibited	Time-lapse microscopy in under agarose cell migration assays	Time-lapse microscopy in under agarose cell migration assays	(105)
PRKAR1A	PKA regulatory subunit	siRNA: Cofilin	fMLF	FPR1	Myeloid leukemia (PLB-985)	No effect	Inhibited	Time-lapse microscopy in under agarose cell migration assays	Time-lapse microscopy in under agarose cell migration assays	(105)
Mst1	Kinase, actin pathway regulator	siRNA: Mst1	CCL21	CCR7	DC (Mono-DC)	No effect	Inhibited	Transwell assays	Time-lapse microscopy	(145)
Pyk2	Kinase, actin pathway regulator	Dom. Neg DNA: PRNK	CCL19 or CCL21	CCR7	DC (Mono-DC)	No effect	Inhibited	Transwell assays	Time-lapse microscopy	(65)
Mek1/2 Erk1/2	Kinases of the MAPK pathway	Inhibitor: UO126	CCL19 or CCL21	CCR7	DC (Mono-DC)	Inhibited	No Effect	Transwell assays	Time-lapse microscopy	(65)
		Inhibitor: PD98059	CCL19	CCR7	DC (BM-DCs)	Inhibited	No Effect	Time-lapse microscopy in microfluidic devices	Time-lapse microscopy in microfluidic devices	(101)
p38	Kinase of the MAPK pathway	Inhibitor: SB203580	CCL19 or CCL21	CCR7	DCs (Mono-DC)	Inhibited	No effect	Transwell assays	Time-lapse microscopy	(65)
JNK	Kinase of the MAPK pathway	Inhibitor: SP600125	CCL19 or CCL21	CCR7	DC (Mono-DC)	Inhibited	No effect	Transwell assays	Time-lapse microscopy	(65)

For additional abbreviations see also legends to **Figure 1** and **Table 2**. Notes: (1) Please note that the Formyl peptide receptor 1 (FPR1) is called generically fMLF-R before it was cloned. BM-Neu, Bone-marrow-derived neutrophils; BPB, PLA2 inhibitor p-bromophenacyl bromide; Cytoch. D: Cytochalasin D; DOCK, dedicator of cytokinesis; Dom. Neg, Dominant Negative; GC (guanylyl cyclases GCA and sGC); FPR1, Formyl peptide receptor 1; GEF; Guanine nucleotide exchange factor; GFP, green fluorescent protein; GFP-RBD, Ras binding domain fused with the GFP; hMδ (human macrophages); IP3, inositol 1,4,5-trisphosphate; Jasplak., Jasplakinolide; JNK, c-Jun N-terminal kinase; Latrun, Latrunculin; mGRANs, murine granulocytes; MAPK, mitogen activated protein kinase; Mono, monocyte; LY, PI3K inhibitor LY294002; Mst1, Mammalian Sterile 20-Like 1; PKCα, Protein Kinase C α; PH, pleckstrin homology; PH-AKT-GFP, PH domain of Akt fused with the GFP; PH-Crac-GFP, PH* *domain of cytosolic regulator of adenylyl cyclase fused with GFP; PI3K, phosphatidylinositol 3-kinase; PI3K2-GFP PI3K2 fused with GFP; PLA2, phospholipase A2; PM, plasma membrane; PRNK, Pyk2-related non-kinase, a dominant-negative Pyk2 construct; Ric8, Resistance to inhibitors of cholinesterase 8; SHIP1, SH-2 containing inositol 5’ polyphosphatase 1; TAT-C3, C3-exoenzyme N-terminus fused with the Tat (trans-activating transcription factor) transduction domain of human immunodeficiency, WASP, Wiskott-Aldrich Syndrome Protein; S1P, Sphingosine-1-phosphate.

Finally, the results obtained also suggest that what best defines functionally a chemoattractant receptor is its ability to govern directional sensing, not cell motility. In this regard, it has been shown that in addition to gradient sensing, chemoattractant receptors can govern a variety of other non-intrinsic cell activities in different contexts, including, survival, endocytosis, changes in cytoarchitecture, neutrophil extracellular trap (NETs) formation, and others (146). We hypothesize that the regulation of chemokinesis could be one of the additional activities governed by these receptors (147), although, probably, after directional sensing, could be the activity more commonly associated with these receptors in most contexts (**Figure 2**).

## Predictably chemoattractant receptors that regulate chemokinesis will also govern regulators of actin-controlled cell motility

**Figure 3:**
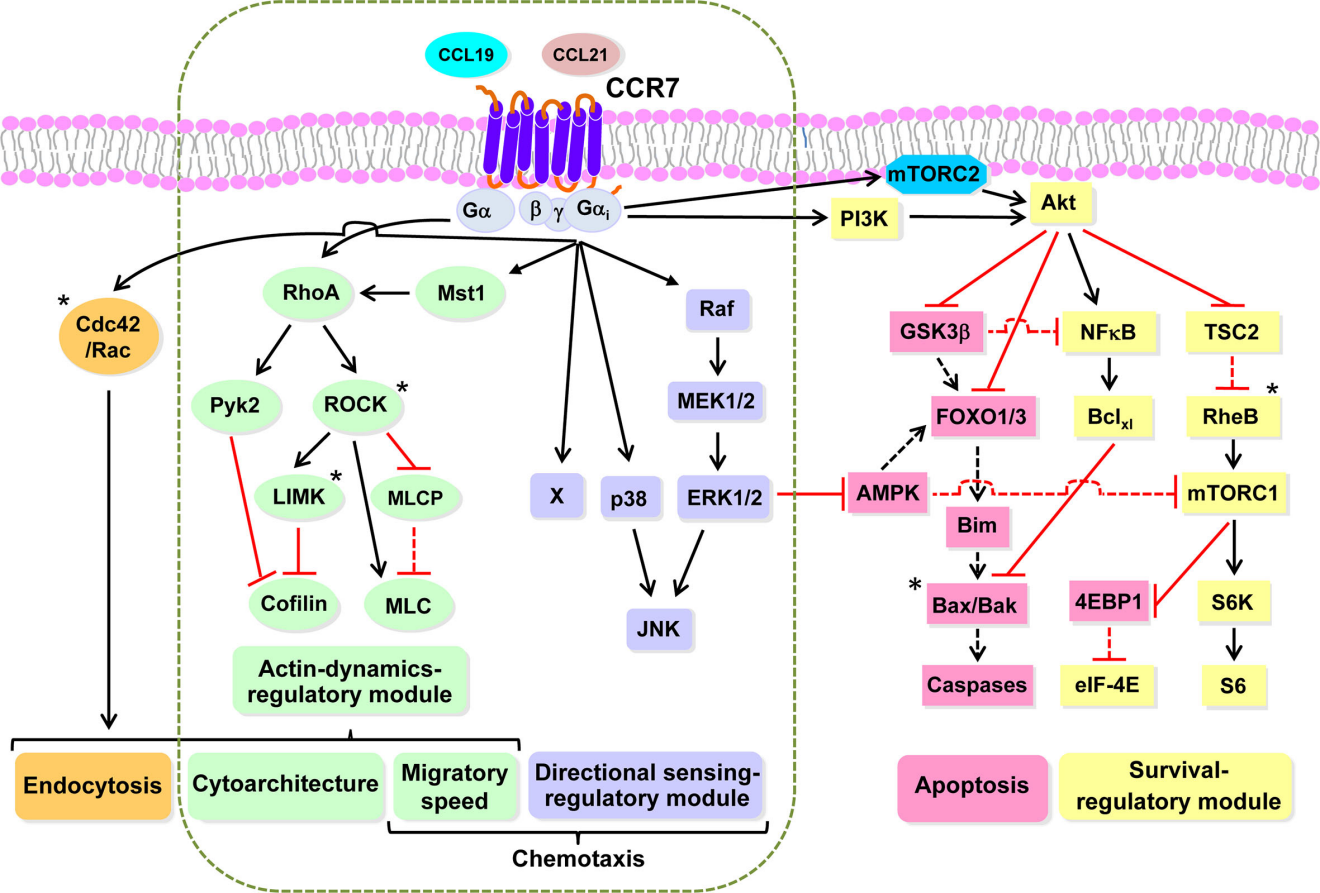
CCR7 uses highly independent signaling modules to regulate the functions of dendritic cells Working model based largely on our experimental data obtained studying the functions and signaling molecules controlling the functions of CCR7 in DCs. Black and red lines indicate stimulatory and inhibitory effects, respectively. A dashed black or red line indicates that after stimulation of CCR7, ceases the indicated effect, either activation or inhibition, exerted by the indicated molecule. An asterisk indicates molecules not analyzed experimentally by us, which are included based on bibliographic information. Endocytosis can be downstream of RhoA (green) and Rac/Cdc42 (orange). The dashed vertical rounded rectangle includes the signaling modules governing CCR7-induced actin dynamics and directional sensing. Signaling module governing CCR7-mediated directional sensing (mallow). Signalling module controlling CCR7-mediated actin dynamics (green). Signaling module controlling CCR7-mediated survival (yellow). Pro-apoptotic molecules that are inhibited by the survival regulatory module (flesh colour). Abbreviations used (see also legend to **Figure 1**, for additional abbreviations): Akt, AK strain mouse Thymoma; AMPK; 5’ AMP-activated protein kinase; Bak, Bcl-2-antagonist/killer; Bax, Bcl-2-Associated X Protein; Bclxl, B-cell lymphoma-extra-large; Bim, Bcl-2 Interacting Mediator of cell death; Mst1, Mammalian sterile 20-like kinase 1; eIF4E, Eukaryotic translation initiation factor 4E; 4E-BP1, eIF4E-binding protein 1; FOXO1/3, Forkhead box O 1/3; GSK3β, glycogen synthase kinase-3β; JNK, c-Jun N-terminal kinase; p38, p38 mitogen-activated protein kinases; MEK1/2, MAPK/ERK Kinase 1 and 2; ERK1/2, extracellular signal-regulated kinase 1 and 2; mTORC1, mTORC2, mechanistic target of rapamycin complex 1 and 2; NFκB, Nuclear factor kappa-light-chain-enhancer of activated BC; PM, plasma membrane; Pyk2, Proline-rich tyrosine kinase 2; Raf, Rapidly accelerated fibrosarcoma kinase; S6, S6 protein; RheB; Ras homolog enriched in brain; S6K, S6 kinase; TSC2, Tuberous sclerosis complex 2.

When analyzing the two types of chemoattractant receptors described above, receptors that do (**Figure 2**) or do not control chemokinesis (**Figure 2**), it can be predicted that only the receptors in the second group will be able to relay intracellular signals that connect with the actin-controlled motile machinery (**Figure 2**). There is some experimental evidence supporting this concept. For instance, the stimulation of neutrophils with fMLF promotes chemokinesis (102, 114, 115, 120) also induces actin polymerization (148). CCR7, which regulates chemokinesis in human mature DCs, also controls actin-based motility (65, 117). In this regard, CCR7 controls the kinase (Mammalian Ste20-like kinase1) Mst1 and the small GTPase RhoA, which are upstream regulators of cofilin, and MLC, which mediates actin turnover and actomyosin-mediated contractility, respectively (65, 145) (See **Figures 2**, **3**). Stimulation of CCR7 also induces chemokinesis in T cells (149–151) and, accordingly, this receptor also induces activation of RhoA, which regulates actin-based motility (152, 153). Stimulation of neutrophils with fMLF (receptor FPR1) (154, 155), or *Dictyostelium* with cAMP (receptor cAR1) (156), induces chemokinesis and promotes actin polymerization in both cell types (154–156). Finally, in line with these results, it can be also predicted that chemoattractant receptors that govern directional sensing, but not chemokinesis (**Figure 2**), will not control the actin-based motile machinery.

## Chemoattractant receptor-mediated directional sensing and actin-controlled motility can be independently regulated

As indicated above, in most published models it is accepted that downstream of chemoattractant receptors, the signaling pathways that regulate directional sensing and motility are intertwined (15, 32–36). If this were the case, then interference with key signaling molecules involved in the control of any of these two functions it would simultaneously affect both of them. However, in a meta-analysis in which we tested this concept, it was found that the selective inhibition of key molecular regulators of either directional sensing or cell motility fail to block simultaneously both functions, suggesting that these two activities could be regulated independently. The results of this metanalysis are summarized in **Tables 3**, **4**, which include data on GPCR and RTK families of chemoattractant receptors, respectively. Below we discuss briefly the results obtained with each one of the molecules shown in the tables.

**Table 4:** Independent regulation of directional sensing and motility downstream of the Receptor Tyrosine kinase family of chemoattractant receptors.

Target protein	Role of target protein	Tool used to block the target	Chemo-attractant	Chemo-attractant receptor (RTKs)	Cellmodel	Effect of inhibition ofthe target on:		Method/device used to analyze		Refs
						Directional sensing	Motility	Directional sensing	Motility	
Rac1	GTPase, actin regulator	siRNA: Rac1	PDGF	PDGF-R	Fibroblasts (MEF)	No effect	Inhibited	Time-lapse microscopy in Dunn chambers	Time-lapse microscopy in Dunn chambers	(71)
Cdc42	GTPase, actin regulator	siRNA: Cdc42	PDGF	PDGF-R	Fibroblasts (MEF)	No effect	Inhibited	Time lapse microscopy in Dunn chambers	Time lapse microscopy in Dunn chambers	(71)
RhoG	GTPase, actin regulator	siRNA: RhoG	PDFG	PDGF-R	Fibroblasts (MEF)	No effect	Inhibited	Time-lapse microscopy in Dunn chambers	Time-lapse microscopy in Dunn chambers	(71)
>Arp2/3 complex	>Actin branching regulator	Knock-out: Arp2/3^-/-^	PDGF	PDGF-R	Fibroblasts (mfibroblasts)	No Effect	Inhibited	Time-lapse microscopy in microfluidic devices	Time-lapse microscopy in microfluidic devices	(68)
		siRNA: Arp2/3	PDGF	PDGF-R	Fibroblasts (IA32)	No effect	Inhibited	Time-lapse microscopy in microfluidic devices	Time-lapse microscopy in microfluidic devices	(88)
		siRNA: Arp2/3	EGF	EGF-R	Fibroblasts (IA32)	No effect	Inhibited	Time-lapse microscopy in microfluidic devices	Time-lapse microscopy in microfluidic devices	(68, 157)
		Inhibitor: CK-666	PDGF	PDGF-R	Fibroblasts (IA32)	No effect	Inhibited	Time-lapse microscopy in microfluidic devices	Time-lapse microscopy in microfluidic devices	(88)
Myosin II	Regulates actin contractility	Inhibitor: Blebbistatin	PDGF	PDGF-R	Fibroblasts (IA32)	Inhibited	No effect	Time-lapse microscopy in microfluidic devices	Time-lapse microscopy in microfluidic devices	(68)
Myosin IIA	Regulates actin contractility	siRNA: Myosin IIA	PDFG	PDFG-R	Fibroblasts (IA32)	Inhibited	No Effect	Time-lapse microscopy in microfluidic devices	Time-lapse microscopy in microfluidic devices	(68)
WASP	Actin polymerization regulator	WASP^-/-^ patient	CSF-1	CSF1-R	MΦ (h-MΦ)	Inhibited	No effect	Time-lapse microscopy in Dunn chambers	Time-lapse microscopy in Dunn chambers	(108)
PKCα	Ser-Thr kinase with many targets	Inhibitor: Gö6976	PDGF	PDGF-R	Fibroblasts (IA32)	Inhibited	No effect	Time-lapse microscopy in microfluidic devices	Time-lapse microscopy in microfluidic devices	(68)
		siRNA: PKCα	PDGF	PDGF-R	Fibroblasts (IA32)	Inhibited	No Effect	Time-lapse microscopy in microfluidic devices	Time-lapse microscopy in microfluidic devices	(68)
PLCγ1	Mediates the production of DAG and IP3	Knock-Out: PLCγ^-/-^	PDGF	PDGF-R	Fibroblasts (MEF)	Inhibited	No effect	Time-lapse microscopy in microfluidic devices	Time-lapse microscopy in microfluidic devices	(68)
		siRNA: PLCγ^-^	PDGF	PDGF-R	Fibroblasts (IA32)	Inhibited	No effect	Time-lapse microscopy in microfluidic devices	Time-lapse microscopy in microfluidic devices	(68)
PLCε	Mediates the production of DAG and IP3	Knock-Out: PLCε^-/^	PDFG	PDFG-R	Fibroblast (MEF)	Inhibited	No effect	Time-lapse microscopy in Dunn chambers	Time-lapse microscopy in Dunn chambers	(158)

For additional abbreviations see also the legends to **Figures 1** and **Tables 2** ,**3**. CSF-1, colony stimulating factor 1; Colony stimulating factor 1-receptor (CSF1-R), IA32, mouse fibroblasts; mfibroblasts, murine fibroblasts obtaine from mouse tails; MEF, Mouse embryo fibroblast; PLCγ1, Phospholipase Cγ1.

CXCR4. As mentioned above, the inhibition of CXCR4 in HSPCs with AMD3100, a potent antagonist of this receptor, blocked directional sensing, but failed to effect the motility of the cells (134). These results suggest that in HSPCs, CXCR4 governs directional sensing, but not tmotility.

**RasG.** When WT or RasG deficient *Dictyostelium* cells transfected with GFP-RBD are exposed to a gradient of cAMP (receptor cAR1), it was observed that the directional sensing activity of these cells, assessed by the mobilization of GFP-RBD to the side of the membrane exposed to the chemoattractant, was inhibited in the RasG deficient cells, but not in the WT control cells. However, the migratory speed was similar in the WT and RasG-deficient cells exposed to the gradient of cAMP (47). The results suggest that in *Dictyostelium* RasG mediates cAR1-controlled directional sensing, but not motility.

**PI3K/PLA2**. The directional sensing ability and the chemokinesis was analyzed both in *Dictyostelium* “GC null” cells (deficient in Guanylyl cyclase A (GCA) and soluble guanylyl cyclase (sGC) genes), and in “GC null” cells treated with the pharmacological agents LY294002 and p-bromo-phenacyl bromide (BPB), which inhibit PI3K and PLA2 activity, respectively. 1 cases the cells displayed similar migratory speed. However, compared to the untreated “GC null” cells, which displayed a normal directional response, the directionality toward cAMP was impaired in the inhibitor-treated “GC null” cells (45). The results, apart from underlining the importance of PI3K and PLA2 in the regulation of directional sensing in *Dictyostelium*, suggest that in “GC null” cells, cAMP receptor-controlled directional sensing and motility are governed by different signaling pathways.

**PI3Kγ**. Neutrophils deficient in PI3Kγ (PI3Kγ^-/-^) display a reduced migratory speed compared to their WT neutrophils, however, both PI3Kγ-deficient and WT neutrophils show a similar directional sensing ability in response to a gradient of fMLF (receptor FPR1) (140). These results suggest that in neutrophils PI3Kγ controls motility, but not FPR1-controlled directional sensing.

**SHIP1**. The SH-2 containing inositol 5’ polyphosphatase 1 (SHIP1) opposes the effects of PI3K because it converts PIP3 into PI (3, 4)P2. It has been observed that neutrophils deficient in SHIP1, show a reduction in their migratory speed when compared to WT controls. However, both WT and SHIP1-deficient neutrophils displayed similar directional sensing ability in response to a gradient of fMLF (receptor FPR1) (140). These results suggest that in neutrophils SHIP1 regulates motility, but not FPR1-controlled directional sensing.

**Ras GEF Aimless.** The directional sensing response of *Dictyostelium* cells exposed to a gradient of cAMP (receptor cAR1) was reduced in cells lacking the Ras-GEF Aimless, but not in the WT cells. However, the migratory speed was similar in the Ras-GEF deficient or the WT cells (141), suggesting that Aimless selectively controls directional sensing, but not migratory speed in this system. The results suggest that in *Dictyostelium* the Ras GEF Aimless mediates cAMP receptor cAR1-controlled directional sensing, but not the motility of these chemotactic cells.

**Rac1.** In response to a gradient of fMLF (receptor FPR1) both WT and Rac1-deficient neutrophils display a similar migratory speed. However, the directional sensing is inhibited in Rac1-deficient neutrophils but not in the WT cells (102). The results suggest that in neutrophils Rac1 mediates FPR1-controlled directional sensing, but not the motility of these cells.

**Rac2.** Rac2-deficient neutrophils show an important reduction in their migratory speed compared to their WT counterpart, however, both Rac2-deficient and WT neutrophil controls display similar directional sensing ability in response to a gradient of fMLF (receptor FPR1) (102). Therefore, in neutrophils Rac2 regulates motility, but not FPR1-controlled directional sensing.

**Cdc42**. Bone marrow-derived DCs (BM-DCs) obtained from control (WT DCs) or Cdc42 deficient mice (Cdc42^-/-^DCs) display in 2D or 3D migration analyses, similar directional sensing ability in response to gradients of CCL19 (receptor CCR7) (142). However, in these experiments it was observed a reduced migratory speeds of the Cdc42^-/-^DCs compared to the WT DCs (142). Hence, in BM-DCs the small GTPase Cdc42 governs motility, but not CCR7-controlled directional sensing.

**RIC8.** The migratory speed is reduced in *Dyctiostelium* cells lacking RIC8, a GEF for Gα proteins, but not in the WT cells. However, both WT and RIC8 deficient cells displayed correct directional responses when they were exposed to a high concentration gradient of cAMP (receptor cAR1), suggesting that in this setting RIC8 mediates motility, but not directional sensing (143). The resuls indidicate that in *Dyctiostelium*, the GEF RIC8 regulates motility, but not cAR1-controlled directional sensing.

**F-actin.** The treatments of BM-Mϕ with the F-actin inhibitor Cytochalasin D induced a reduction of the motility of the cells, however, F-actin disruption does not affect the ability of these cells to detect a gradient of C5a (100). Likewise the treatment of BM-DCs with the actin organization inhibitor Latrunculin A also leads to a reduction of their migratory speed; however, their ability to migrate in the direction of a gradient of the chemokine CCL19 was not altered (101). In studies performed in *Dictyostelium* cells transfected with the fluorescent biosensor Ras Binding Domain (RBD)- GFP treated or not with the actin disrupting drugs Latrunculin A, and then exposed to a gradient of cAMP, it was observed that in the Latrunculin A-treated cells the motility was reduced compared with the untreated controls (47). However, RBD-GFP increased in the membrane of the cells exposed to a gradient of cAMP (47), suggesting that the directional sensing machinery was not affected. Similar results were obtained with *Dictyostelium* cells transfected with either of two different PH-domain-containing fluorescent biosensor probes, namely Cytosolic regulator of adenylyl cyclase (Crac)-GFP or phosphatidylinositol 3-kinase 2 (PI3K2)-GFP, which, as indicated above, detect PIP3 at the membrane. The transfected cells were treated or not with Latrunculin A or Cytochalasin A, to disrupt F-actin, and then exposed to a gradient of cAMP. In these experiments it was observed that the motility of the Latrunculin A- or Cytochalain A-treated cells was clearly inhibited, however, their directional sensing ability, as indicated by the translocation of the fluorescent bioprobes to the side of the membrane exposed to the gradient of cAMP, was not altered (44, 47, 56, 59, 144). Finally, additional experiments were performed with HL-60 neutrophils transfected with the biosensor PH Akt1 (PH-AKT), which binds to PIP3. When the transfected cells were pre-treated with Latrunculin B, to depolymerize F-actin, and subsequently the cells were exposed to a gradient of fMLF, an increase of PH-AKT was observed at the site of the cells exposed to the gradient of this chemoattractant, suggesting a correct functioning of the directional sensing molecular machinery in these cells (43). Please note that the authors did not measure the motility of the cells in the latter study (43), however, multiple studies show that the disruption of F-actin with Latrunculin B lead to a partial or total reduction of the motility of the cells (see above). Together these experiments suggest that directional sensing does not require F-actin organization.

**RhoA.** Experiments to study the role of this small GTPase were carried out either with human mono-DCs in which RhoA was inhibited with the C3-exoenzyme (65) or, alternatively, with monocytes in which RhoA was inhibited with TAT-C3, a membrane-permeating form of the C3-exoenzyme. TAT-C3 consists of the C3-exoenzyme fused with the Tat (trans-activating transcription factor) transduction domain of human immunodeficiency virus (109). When, the C3-exoenzyme-treated DCs were exposed to a gradient of CCL19 or CCL21 (receptor CCR7) (65) or when the TAT-C3-treated monocytes were exposed to a gradient of fMLF (receptor FPR1), it was observed that compared to the untreated controls, the motility of the inhibitor-treated DCs and monocytes was suppressed. However, in these experiments it was also observed that the directional sensing ability of the DCs, in response to gradients of CCL19 or CCL21 (65) or the monocytes, in response to fMLF (109), was not affected. The results suggest that in DCs (human monocyte-derived-DCs) and monocytes the small GTPase RhoA regulates motility, but not CCR7 or FPR1 mediated directional sensing.

**GEF-H1 (ARHGEF2).** The reduction with siRNA of this Rho GEF in myeloid leukemia cells resulted in reduction of the motility of the cells, but not in their directional response in response to a gradient of fMLF (receptor FPR1) (105). Therefore, in myeloid leukemia cells GEF-H1 regulates motility, but not FPR1-regulated directional sensing.

**ROCK**. BM-DCs that were treated with the ROCK inhibitor Y27632, displayed a similar ability to detect gradients of CCL19 or CCL21 in migration studies carried out on 2D surfaces or in 3D analysis in collagen gels (80, 101, 110). However, under these conditions the DCs treated with Y27632 displayed a reduced migratory speed (80, 101, 110). Likewise, in chemotaxis analyzes in 3D collagen gels performed with murine granulocytes (GRAN) and BCs, treated or not with Y27632, and subsequently exposed to a gradients of CCL19, it was also observed inhibition of migratory speed of the Y27632-treated cells, but not in the ability of these cells to detect the direction of the chemokine (80). Similarly, in 3D collagen gel analysis carried out with human monocytes (109) and murine BM-Mϕ (100), which were treated with Y27632, the inhibitor-treated cells displayed a reduced motility, when compared with the motility of untreated control cells. However, both Y27632-treated and untreated monocytes (109) were able to detect gradients of fMLF (receptor FRP1) and Y27632-treated and untreated BM-Mϕ (100) were also able to detect similarly gradients of the chemoattractant C5a (receptor C5aR). The results indicate that in BM-DCs, in BCs, and in GRANs, the kinase ROCK regulates motility, but not CCR7-regulated directional sensing. Furthermore, both in monocytes and macrophages (BM-Mϕ), ROCK regulates motility, but not FPR1-mediated directional sensing in monocytes or C5aR-mediated directional sensing in BM-Mϕs.

**F-Actin/ROCK.** When the neutrophilic cell line HL-60 transfected with PH-AKT were treated with a mixture of the actin disassembly inhibitor Jasplakinolide, the actin polymerization inhibitor Latrunculin A, and the ROCK inhibitor Y27632 (51), it was observed that the cells remained immobilized on the substrate and their shape was preserved (51). When these immobilized neutrophils were exposed to a gradient of fMLF (receptor FPR1), PH-AKT increased in the surface of the cells facing the chemoattractant, indicating that directional sensing machine was functional, despite the fact that the cells were completely immobilized. Therefore, in neutrophils the kinase ROCK and F actin regulate motility, but not FPR1-dependent directional sensing.

**Myosin II**. BM-DCs treated with the Myo II inhibitor Blebbistatin displayed similar directional sensing response on 2D motility analysis, and in 3D analyzes carried out with cells included in collagen gels. On the other hand, the directional sensing ability of these cells in response to CCL19 or CCL21 was not altered (80, 101, 110). Similarly, 3D analyzes performed with BCs or GRANs, treated or not with Blebbistatin, in response to a gradient of CCL19, showed that the Blebbistatin-inhibited cells displayed a reduction in their migratory speed, however, their directional sensing response was not affected by the inhibition of Myo II (80). Finally, similar results were obtained when the migration of Myo II-deficient DCs (Myo II^-/-^) was analyzed in 3D collagen gels. In these experiments, it was observed that in Myo II-deficient DCs the motility was reduced compared with the WT DCs, however their directional response to CCL21 was not altered (110). Hence, in BM-DCs, in BCs and GRANs, the small GTPase RhoA regulates motility, but not CCR7-mediated directional sensing.

**mDia.** In 3D motility studies carried out with mature BM-DCs embedded in collagen gels, it was observed that the reduction of the actin-associated protein mDia inhibited the motility of these cells, but not their directional response toward CCL21 (106, 111). The results indicate that in DCs mDia regulates motility, but not CCR7-controlled directional sensing.

**WASP.** Neutrophils in which the actin cytoskeleton regulator WASP was knock-down with short hairpin RNAs (shRNAs), displayed a similar directional response toward a gradients of fMLF both in the WASP deficient and the WT control neutrophils, however the WASP deficient cells displayed a reduction in their migratory speed compared with WT control cells (107). Therefore, in neutrophil WASP governs motility, but not FPR1-controlled directional sensing.

**WAVE.** BM-DCs deficient in the nucleation promoting factor WAVE (obtained through deletion of Hem1, a subunit of the WAVE complex (159)), displayed a reduced 3D motility in collagen gels, however, their ability to detect the direction of a gradient of the chemokine CCL19 was not altered (112). Hence, in DCs, WAVE governs motility, but not CCR7-mediated directional sensing.

**DOCK2.** DOCK2-deficient neutrophils (103) or T cells (104) displayed a reduction in their migratory speed compared with WT neutrophil and T cells, respectively. When the gradient sensing ability of these cells was examined, in the case of the neutrophils, it was observed that the ability of DOCK2-deficient and WT neutrophils to detect the direction of gradients of the chemoattractants fMLF (receptor FPR1) or C5a (receptor C5aR) was not altered (103). In the case of the T cells, it was similarly observed that the DOCK2-deficient and WT T cells showed a similar directional sensing response to gradients of sphingosine-1-phosphate (S1P) (receptor S1P-R) (104). The results suggest that in neutrophils and T cells DOCK2 regulates motility, but not FPR1- or C5aR-dependent directional sensing in neutrophils or S1P-R mediated directional in T cells.

**Cofilin/Slingshot/Profilin/RHGEF2/PRKAR1A.** The analysis of the response of the myeloid leukaemia cell line PLB-985 to the chemoattractant fMLF (receptor FPR1) showed that the knocking down of the actin-binding proteins cofilin, the cofilin phosphatase slingshot, the cytoskeletal regular profilin, and the Protein Kinase CAMP-Dependent Type I Regulatory Subunit Alpha (PRKAR1A) led in all cases to inhibition of the motility of the cells. However, when PLB-985 control cells, with normal levels of these proteins, and PLB-985 cells in which these molecules were knocked-down, were exposed to gradients of fMLF it was observed that all the cells displayed similar directional sensing ability (105). Therefore, in leukaemia cells cofilin/slingshot/profilin/RHGEF2/PRKAR1A regulate motility, but not FPR1-dependent directional sensing.

**Mst1** The inhibition of the kinase Mst1 in the mature human DCs with siRNA affected the motility of the cells but not the response of the cells to a gradient of CCL21 (145). The results indicate that in DCs the kinase Mst1 governs motility, but not CCR7-controlled directional sensing.

**Pyk2.** The inhibition of the tyrosine kinase Pyk2 with a dominant-negative DNA in mature human DCs led to a reduction in the migratory speed of the CCR7-stimulated DCs, however, the directional sensing response of the cells toward CCL21 was not altered (65). Hence, in DCs the kinase Pyk2 governs motility, but not CCR7-controlled directional sensing.

**MEK1/2/ERK1/2, p38 and JNK**. When in DCs (human mono-DCs or murine BM-DCs), the MAPK members MEK1/2/ERK1/2 were inhibited with UO126 or PD98059, the kinase p38 was inhibited with SB203580, and c-Jun N-terminal kinase (JNK) was inhibited with SP600125, it was observed that the ability of the cells to detect the direction of CCL19 or CCL21 was inhibited, but not the migratory speed of the cells that was not altered (65, 101). The results indicate that in DCs, MEK1/2/ERK1/2, p38 and JNK govern motility, but not CCR7-controlled directional sensing.

Finally, further suggesting that GPCR-mediated directional sensing and motility are independently regulated, recent experiments show that these two activities are not similarly affected by biological stimuli that modify dramatically the physiology of the cells. In this regard, long-term treatment of DCs with lipopolysaccharides (LPS) leads to a condition called exhaustion, in which the ability of leukocytes to produce cytokines is greatly reduced (160). Interestingly, when both untreated controls and exhausted DCs were exposed to a gradient of CCL19 (receptor CCR7), the migratory speed of the exhausted cells was reduced compared with the untreated controls, however, the directional sensing ability was similar both in control and exhausted DCs (161). If the signalling molecules that regulate both activities would have been interconnected, it would have been expected that both functions should be similarly affected.

Experimental data suggesting independence between the signal pathways controlling directional sensing and motility has also been obtained in studies performed on RTK family of chemoattractant receptors. These receptors are predominantly expressed in cells that display a mesenchymal type of movement, like e.g. fibroblasts (**Table 4**).

**Rac, Cdc42, RhoG**, Transient depletion of any of these three small GTPases, which are regulators of the actin cytoskeleton, in mouse embryo fibroblasts (MEFs), resulted in a reduction of the migratory speed of these cells; but not their ability to detect the direction of the chemoattractant platelet-derived growth factor (PDGF) (receptor PDGF-R) (71). Therefore, in MEFs the small GTPases Rac, Cdc42, Rhogovern motility, but not PDGF-R-controlled directional sensing.

**Arp2/3.** Mouse fibroblasts deficient in Arp2/3 (Arp2/3^-/-^) (68), IA32 fibroblasts in which the actin regulator Arp2/3 was knock-down with siRNA (88), or Rat2 fibroblasts in which Arp2/3 was blocked with the inhibitor CK-666 (88), displayed in all cases a reduced migratory speed. By contrast, the directional response of these fibroblasts to gradients of PDGF was not altered (68, 88). Similar results were obtained when IA32 fibroblasts in which the actin regulator Arp2/3 was knock-down with siRNA, were exposed to a gradient of Epidermal Growth Factor (EGF) (157). Therefore, in fibroblasts the Arp2/3 complex governs motility, but not PDGF-R or EGF-R-controlled directional sensing.

**Myosin IIA**. When fibroblasts treated with Blebbistatin to inhibit Myo II were exposed to a gradient of PDGF, it was observed that their directional sensing was inhibited, but not their motility (68). In other experiments siRNAs were used to reduce the two isoforms of Myosin II expressed in fibroblast, namely Myosin IIA (Myo IIA), and Myosin IIB (Myo IIB). The knocking down of Myo IIB, failed to affect the motility of the fibroblasts or their ability to recognize the direction of a gradient of PDGF. Nevertheless, the reduction of Myo IIA, led to the inhibition of the directional sensing of the fibroblasts in response to a gradient of PDGF, but not their motility. The results indicate that in fibroblasts, Myo IIA governs PDGF-R-controlled directional sensing, but not the motility.

**WASP**. In human macrophages obtained from Wiskott-Aldrich syndrome (WAS) patients that were deficient in WAS proteins (WASP), it was observed that their directional sensing ability in response to gradients of Colony stimulating factor-1 (receptor CSF1-R) was inhibited, although the migratory speed of the cells was not altered (108). Thus, in human macrophages WASP governs CSF1-R-controlled directional sensing, but not motility.

**PKCα.** Studies on the role of protein kinase Cα (PKCα) in the regulation of PDGF-R mediated directional sensing and motility were performed in fibroblasts (IA32 line). In these cells PKCα was inhibited either treating the cells with the pharmacological agent Gö6976, or knocking-down PKCα with siRNA. The experiments performed showed that the inhibition of PKCα blocked the directional sensing ability of the fibroblasts in response to gradients of PDGF, but not the migratory speed of these cells (68). Hence, in fibroblasts PKCα mediates PDGF-R-induced directional sensing, but not motility.

**PLCγ.** In fibroblast in which phospholipase Cγ (PLCγ) was knocked down with siRNA or in PLCγ deficient MEF cells (PLCγ^-/-^), it was observed that in the fibroblasts where PLCγ was reduced, the directional sensing ability of the cells in response to a gradient of PDGF was blocked, however the migratory speed of the cells was not altered (68). Therefore, in fibroblasts PLCγ mediates PDGF-R-induced directional sensing, but not motility in neutrophils.

**PKCε.**In fibroblasts (MEF) deficient in PKCε (PLCε^-/-^) was observed that their directional sensing ability in response to gradients of PDGF was blocked, but not their migratory speed that apparently even increased slightly (158). Accordingly, in fibroblasts PKCε mediates PDGF-R-induced directional sensing, but not the motility.

In summary, the analysis of a variety of chemoattractant receptors belonging to the GPCR or RTK families, expressed in different cell types, show that the selective inhibition of signaling regulators of either directional sensing or motility fails to affect both functions simultaneously. These results support that these two activities are regulated by independent signaling pathways.

The chemoattractant receptors of the GPCR and RTK families analysed above are mostly expressed in cells that display mesenchymal and amoeboid types of motility, respectively (see above) (72–74). Most studies on chemotaxis have largely focused on chemoattractant receptors of the GPCR family, and the information currently available on RTK family is very sparse. As shown in **Table 2**, the RTK-family members analysed (VEGF-R, VEGF-R2, and PDGF-R), fail to regulate chemokinesis in the cells analyzed (murine bone marrow mononuclear cells (BM-MCs), neural progenitors), implying that these receptors largely govern gradient sensing, but not motility. Additional studies with more TK receptors in different cell types are necessary to determine whether they control exclusively directional sensing or they can also regulate chemokinesis in cells.

We analyzed in **Tables 3**, **4**, which include GPCRs and RTKs, respectively, whether both types of receptors use similar mechanisms to regulate chemotaxis. We analyzed whether a set of signaling molecules that relay signals from both types of receptors (see Cdc42, WASP, Myo II, and Rac1 in **Tables 3**, **4**), could affect GPCR- and RTK-mediated directional sensing and motility. Only Cdc42, which regulates motility, but not directional sensing, exerts a similar effect downstream of both types of receptors (71, 142). However, Rac1, WASP and Myo II, play opposite roles downstream of GPCRs and RTKs. In this regard, while WASP controls directional sensing, but not motility, in macrophages that express the RTK CSF1-R (108); however, this actin polymerization regulatory molecule governs motility, but not directional sensing, in neutrophils that express the GPCR FPR1 (107). Myo II mediates directional sensing, but not motility, in fibroblasts that express the RTK PDGF-R (68); nevertheless, this regulator of actin contractility, governs motility, but not directional sensing, in DCs, BCs, and GRANs that express the GPCR CCR7 (80, 101, 110). Finally, Rac1 mediates motility, but not directional sensing in fibroblasts that express the RTK PDGF-R (71); however, this small GTPase controls directional sensing, but not motility, in neutrophils that express the GPCR CCR7 (102). Hence it seems that, regarding the regulation of directional sensing and motility, in general, GPCRs and RTKs use the same signaling molecule to regulate opposite activities. As indicated previously by other authors the results suggest that chemoattractant receptors of the GPCR and RTK families use different signaling pathways to regulate directional sensing and motility (68). Recently it has been suggested that the axis PLCγ-PKCα-Myo IIA (68), plays a key role in RTK-controlled directional sensing (**Table 4**), while the GPCR-mediated chemotaxis seems to involve several alternative pathways (**Table 3**) (34, 45).

## The signaling components governing CCR7-controlled directional sensing and actin-mediated motility may constitute independent signaling modules in dendritic cells

It has been suggested that modularity could be a key principle of the signaling pathways (162). An interesting question is to determine whether the existence of independent signaling modules could explain the separation between directional sensing and chemokinesis downstream of chemoattractant receptor in chemotactic cells. In this regard, the existence modularity is consistent with the abundant data discussed above (**Tables 3**, **4**), showing that the selective inhibition of key signaling components governing directional sensing or motility downstream of a variety of chemoattractant receptors fails to affect the other activity (147) (**Figure 3**). Although this is an issue that deserves to be analysed further in different chemoattractant receptors, studies carried out on the components of the pathways that mediate CCR7-controlled directional sensing and chemokinesis in DCs suggest that modularity could be a potential mechanism to separate these two pathways (65, 101, 110, 111, 145, 147).

We have reported before that CCR7 governs in DCs two independent signaling modules that may control directional sensing and chemokinesis (**Figure 3**). We found that CCR7-governed directional sensing is governed by the axis Gi-Raf-MEK1/2-MAPK kinases (ERK1/2, JNK, and p38 and an unidentified component that we call X) (65, 147). CCR7-governed chemokinesis is governed by the axis G_i_-G_12/13_-Mst1-RhoA (145, 147). We call the signaling module that controls cell motility “actin dynamics regulatory module”. As most chemotactic cells display spontaneous motility before they are stimulated by chemoattractants, we think that the “actin dynamics regulatory module” can be a preexistent signaling module with which CCR7, like other chemoattractant receptors that control chemokinesis, can connect through the G_i_-G_12/13_ proteins. Downstream of CCR7, RhoA controls F-actin dynamics through the activation of mDia, which, as indicated above (**Figure 1**), promotes elongation of linear F-actin (111), and the ROCK-LIMK axis mediates phosphorylation/inhibition of cofilin, an actin-binding molecule that in its active state severs and depolymerizes F-actin (163). The RhoA pathway can also promote actomyosin contractility thorough ROCK-mediated phosphorylation/inhibition of MLCP and phosphorylation/activation of the MLC, resulting in the increase in phosphorylated MLC and contractility (101, 111) (**Figure 3**). By contrast G_12/13_ seems to connect CCR7 only with the actin-mediated motile machinery (145). Importantly, selective interference with signaling components involved in either CCR7-governed gradient sensing or motility pathways fails to affect the other pathway (65, 101, 110, 111, 145), suggesting that these two CCR7-mediated activities are controlled by independent signaling modules.

**Figure 4:**
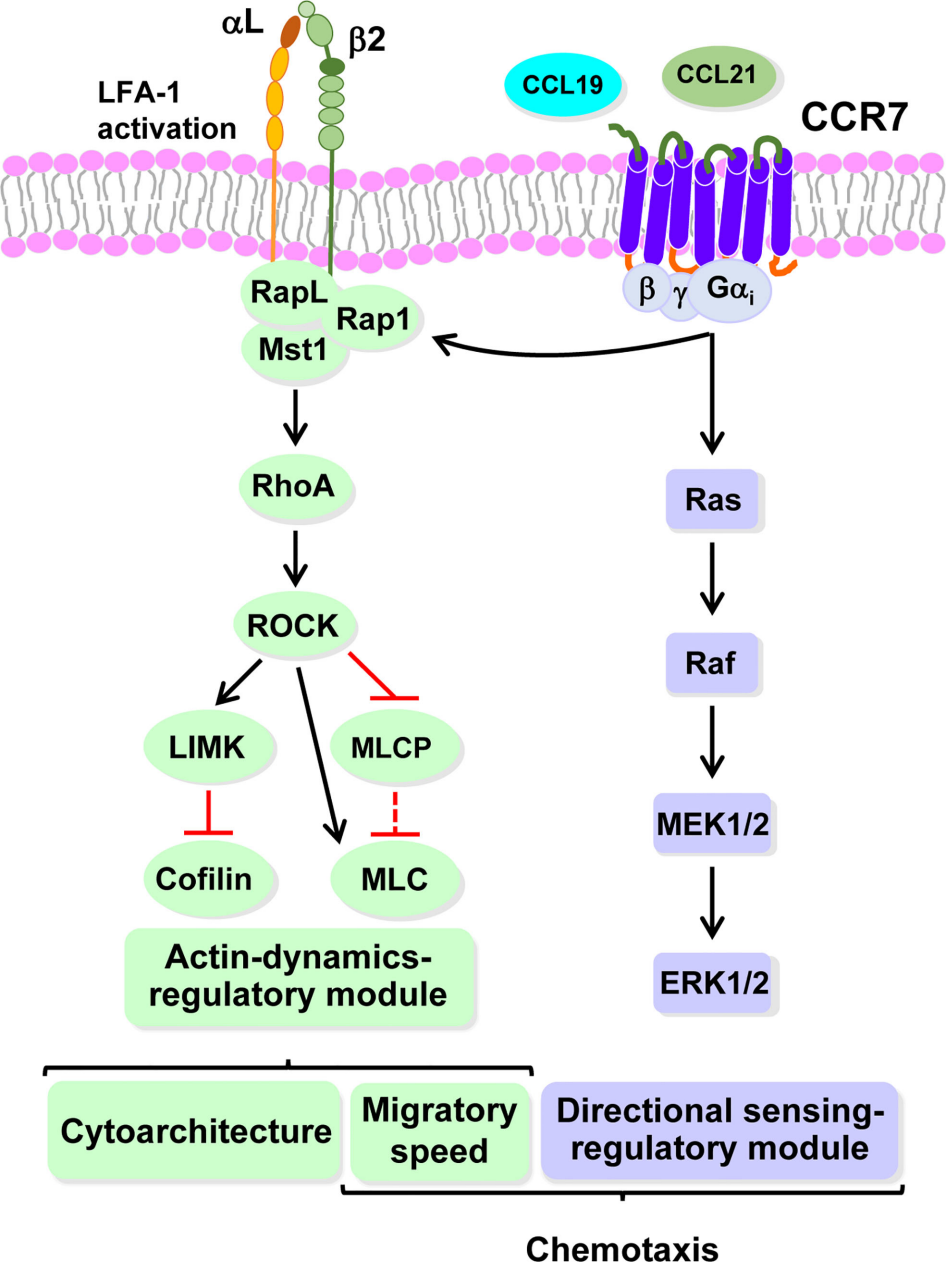
CCR7 could use highly independent signaling modules to regulate directional sensing and migratory speed in T cells. Hypothetical model based on recently published data (see text for details). The signalling module controlling CCR7-mediated actin dynamics that governs cytoarchitecture and migratory speed is downstream of RhoA, which is regulated by Mst1/RapL/Rap1 (green). RapL/Rap1 also govern LFA-1 integrin activation. Signaling module governing CCR7-mediated directional sensing (mallow). Abbreviations (see legends to **Figure 1**; **Tables 2**, **3**, for additional abbreviations): LFA-1 (αlβ2), Lymphocyte function-associated antigen-1; Rap 1, Ras-related protein 1; Rap L, Rap1-binding molecule, regulator of adhesion and cell polarization enriched in lymphoid tissues; Ras, Rat sarcoma viral oncogene homolog.

It is interesting to determine whether CCR7-dependent signaling modules controlling directional sensing and motility is exclusive of DCs or may be used by other cell types. The analysis of published data on the signaling components use by CCR7 to regulate directional sensing and chemokinesis in T cells, suggests that CCR7 may also use signaling modules to control these functions in T cells (149–151, 164–166) (**Figure 4**). Stimulation of CCR7 in T cells leads to rapid Gi-dependent activation of the small GTPase Rap1, which controls changes in actin cytoarchitecture and chemokinesis (149–152, 166). Rap1 and RapL also regulate the activation of the integrin LFA-1 (αLβ2). In addition, these two small GTPases mediate the activation of the kinase Mst1, which is upstream of RhoA and controls T cell chemokinesis (152, 153, 167). As indicated above, RhoA governs molecular targets involved in the regulation of actin polymerization through the axis ROCK-LIMK-cofilin, and contraction, through the axis ROCK/MLCP/MLC (168). On the other hand, stimulation of CCR7 in T cells also induces activation of Ras/MEK1/2/ERK1/2. Inhibition of MEK1/2/ERK1/2 blocks CCR7-induced directional sensing in T cells, suggesting that this pathway selectively regulates this arm of chemotaxis in these cells (169, 170). Hence, the experimental data gathered suggest that it is possible that downstream of CCR7 in T cells Gi-Rap1-RapL-Mst1-RhoA and Ras-MEK1/2-ERK1/2 axes could govern chemokinesis and directional sensing, respectively (**Figure 4**). An important issue is the molecular mechanism that could maintain separated the signaling modules regulating directional sensing and chemokinesis. It has been postulated that directional sensing could be governed at the leading edge, in a process in which even filopodia may also play a role (37, 38, 171). On the other hand, it has been shown that migratory speed can be regulated by MyoII-actin based contractility (68, 101, 110). (**Tables 2**, **3**), which is known to be necessary for the retraction of the trailing edge in the motile cells (32, 111, 172). Therefore, the signaling modules regulating directional sensing and chemokinesis could be selectively located at opposite regions in the chemotactic cell. In summary, more experimental data is necessary to determine whether chemoattractant receptors could use independent signaling modules to govern directional sensing and motility and the mechanisms that govern this process. In summary, the multiple examples supporting that directional sensing and motility are independently regulated in different cell types and different receptors (**Tables 3**, **4**), suggest that many receptors may also use independent signaling modules to control these two activities.

## Biological significance of the independent control of directional sensing and cell motility

What could be the biological significance of the independent control of chemokine receptor-mediated directional sensing and motility (chemokinesis)? First, it may reflect the fact that chemoattractant receptors can regulate different activities of cells, including, directional sensing and motility, using independent signaling modules (147). Second, we suggested above that the ability of a chemoattractant receptor to promote chemokinesis could be context-dependent. If this were the case also *in vivo*, this activity could be activated or switched off, without affecting directional sensing. Hence, the independence of these two activities would allow a chemoattractant receptor to adapt the motile behavior of a chemotactic cell according to the context where is migrating at a certain moment (see below). Third, we also hypothesize that the independent control of directional sensing and chemokinesis makes the migration of chemotactic cells more resistant to alterations in individual signaling components of any of these two pathways. In this regard, if the molecular components controlling both activities were interconnected, it would be expected that the alteration of a single molecule governing either directional sensing or motility would simultaneously affect both of them, and the impact on the migration of the cells would be greater.

An interesting point, related to the issue of the independence of directional sensing and motility, is to determine the specific role of chemokinesis in the organism. Predictably chemokinesis could be important for cells that have to migrate rapidly to their specific targets. We tested this prediction by analyzing the ability of chemokine receptors to control chemokinesis in innate cell, because they have to migrate rapidly to the inflammatory regions to remove threats to the organism. Neutrophils are the first innate leukocyte populations that arrive at the inflammatory sites, where they can actively kill bacteria and other pathogens (173). Interestingly, neutrophils express a variety of inflammatory receptors that induce chemokinesis, including, CCR1/CCR5 (114, 115), CXCR1/CXCR2 (118–120), the fMLF receptor (102, 114, 115, 120), and the complement component 5a receptor (C5a-R) when it is presented to the cells at a low, but not a high concentration gradient (30). The natural killer (NK) cell is another innate cell that can kill tumor and virus-infected cells. Although they arrive later than neutrophils at the inflammatory sites, some of the inflammatory receptors expressed by NK cells including CCR2 and CCR1/CCR3/CCR5 (113) also regulate chemokinesis. Hence, chemokinesis promoted by inflammatory receptors in neutrophils and NK may contribute to the rapid migration of these cells to the inflammatory sites.

During chronic inflammatory processes, including autoimmune diseases like rheumatoid arthritis, different populations of leukocytes, including macrophages, monocytes, and lymphocytes expressing inflammatory chemokine receptors, continuously migrate to inflamed regions contributing to perpetuating the process (174–176). The induction of chemokinesis by inflammatory receptors in these leukocytes may also contribute to their rapid migration to these regions. In this regard, the components of the signaling pathways regulating directional sensing and motility could be interesting targets to curb leucocyte migration in chronic inflammatory diseases (175, 176). Finally, it is important to state that chemokinesis has been largely observed *in vitro*. Recent studies have confirmed that in T cells and DCs, CCR7 also promotes chemokinesis *in vivo* (116, 177). However, in general, the determination of the contribution of chemoattractant receptor-controlled chemokinesis to the response of the leukocytes *in vivo* awaits to be experimentally addressed. One additional complication that can be anticipated in future studies on the role of chemokinesis *in vivo*, is the observation that chemoattractant receptors regulate other cellular activities, not only directional sensing and chemokinesis (146). Hence, for instance, to target selectively chemokinesis in leukocytes, to analyze its role during the immune response, will be necessary to obtain first detailed information on the molecular mechanism used by chemoattractant receptors to govern selectively chemokinesis in the specific cell type analysed.

## Discussion

In this article, we focus on the ability of chemoattractant receptors to govern directional sensing and motility, with an emphasis on motility. We have re-examined two important features of the current models describing the functions controlled by chemoattractant-receptors (15, 32–36): *first*, the concept that chemoattractant receptors intrinsically govern both directional sensing and motility (chemokinesis), and *second*, the concept that the signaling pathways controlling both activities are intertwined (15, 32–36). Regarding the first point, our meta-analysis indicates that although most chemoattractant receptors control directional sensing they can be functionally subdivided into two groups. Namely, a first group, which includes receptors that control both directional sensing and motility (chemokinesis) and a second group, that includes receptors that control directional sensing, but not motility, i.e. their stimulation does not modify the speed of the cells that migrate toward a chemoattractant (**Tables 1**, **2**). Importantly, the experimental data gathered also suggest that the ability of a chemoattractant receptor to govern chemokinesis could be context-dependent. The data also predicts that the chemoattractants receptors that are able to connect with the signaling components that govern actin-based motility will also control chemokinesis. That is the reason why to get further insight into the signalling components that govern directional sensing is preferable to focus on receptors that control this activity, and not chemokinesis. Focusing on this type of receptors may facilitate a more selective analysis of the components that mediate chemoattractant-receptor governed directional sensing,

From the data gathered also emerges the concept that the ability to govern directional sensing is the activity that best defines a chemoattractant receptor. We suggest that the regulation of the migratory speed (chemokinesis), could be another of the additional non-intrinsic activities that can be governed by these receptors in different contexts, like e.g. cytoarchitecture, endocytosis, survival, cytokine secretion, and others (146). Since according to the well-established definition, chemoattraction involves both directional sensing and motility and, as indicated above, they are two different cell activities, this implies that what is called chemoattraction is in fact, a bifunctional activity. In this connection, it is not correct to state that the stimulation of a chemokine receptor stimulates chemotaxis and motility, because this expression is redundant (see **Figure 3**). When analysing the effects of a specific treatment on chemotactic receptor-induced chemoattraction, it is better to refer to the effects of this treatment on directional sensing and motility.

Chemotactic cells can express several chemoattractant receptors, which allows them to respond to multiple chemoattractants. During their life cycle, these cells can transit through a complex medium, like e.g. inflammatory sites, where they can be simultaneously exposed to several chemoattractants. In this context, it is relevant to ask how different chemoattractant receptors expressed by a single cell may affect the control of directional sensing and motility. Since as indicated in this article, experimental data indicate that cell motility (spontaneous or regulated by receptors, i.e. chemokinesis) can be governed independently of directional sensing, we analyze each of these two activities separately.

Concerning the regulation of motility, studies carried out previously in DCs indicate that actin-based motility may be controlled by an independent signaling module, which we call “actin dynamics-regulatory module” (65, 145, 147) (see some components of this module in **Figures 2**, **3**). As indicated above, we hypothesize that this module governs spontaneous motility in unstimulated cells. In contexts, in which several of the chemoattractant receptors of a cell are simultaneously stimulated, the final speed of this cell will depend on the number of receptors stimulated that induce chemokinesis. If none of the stimulated receptors control chemokinesis, then the cells will keep their basal motility. If the receptors stimulated are able to induce chemokinesis, these receptors, as indicated above, will relay intracellular signaling that will activate the “actin-dynamics controlling module”. Within the limits of the ability of the “actin dynamics regulatory module” to govern the speed of the cells, predictably this speed will increase as the number of receptors that promote chemokinesis raises.

Regarding directional sensing, we have also proposed that chemoattractant receptor-controlled directional sensing could be governed by a specific signaling module in chemotactic cells (147). What would be the effect of the simultaneous stimulation of several chemoattractant receptors on directional sensing? Prior results, obtained in the study of the signaling components governed by CCR7 (147) and CXCR4 (132) in DCs, suggest that the signaling modules that control directional sensing may display different molecular architectures. In this regard, to regulate direction sensing, CCR7 uses in DCs an independent module that includes Gi-MAPK (ERK1/2-JNK-p38) (147). However, in these cells CXR4 uses two signaling axes that converge in the kinase mTORC1: CXCR4-PI3K α/γ/δ -Akt-mTORC1 and CXCR4-PI3Kα/γ/δ-MEK1/2-ERK1/2-mTORC1 (132). Interestingly, it has been suggested that chemotactic cells can integrate signals from multiple chemoattractants and reach their targets efficiently (7, 178, 179). These cells can achieve this feat by migrating sequentially, using in each step a hierarchy of dominant chemoattractant receptors that recognize chemoattractant gradients, that behave as intermediate chemotactic cues, to get the cell closer and closer to its target, until, a last dominant receptor recognizes a chemoattractant gradient, that serves as an end-target cue, which guides the cell to its final destination (7, 178–180). In this elegant model, it is also assumed that each receptor has to become desensitized to allow an effective migration of the chemotactic cell toward the next dominant receptor (7, 179). We propose that during this stepwise process, each one of the dominant receptors guiding the leukocytes to their final target, will form an independent signaling module to control directional sensing before becomes desensitized.

An interesting question is why has been largely accepted that chemoattractant receptors constitutively regulate both directional sensing and chemokinesis. A possibility is that it could be due to fact that there are very abundant the receptors that control chemokinesis. In this regard, most of the receptors whose study has provided the information to build the most models presenting the signaling pathways from chemoattractant receptors, including the cAMP and folic acid receptors in *Dictyostelium*, and the CXCR8 and fMLF receptors in neutrophils (**Table 2**) regulate chemokinesis (125–128, 181–183). Considering the importance of these models, it is possible that many scientist, and mostly non-specialist in the field of chemotaxis, may have assumed that all chemoattractant receptors have the ability to control motility (chemokinesis).

Regarding the second point mentioned above, the data presented above show that the selective inhibition of key regulators of directional sensing or alternatively the inhibition of the motility, does not inhibit simultaneously both functions. These results indicate that directional sensing and actin-based motility (receptor-controlled chemokinesis or spontaneous motility) can be controlled by independent signalling pathways. (**Tables 3**, **4**). This independence between directional sensing and motility is observed both in GPCR and RTK, and it is conserved through evolution because it is observed in *Dictyostelium*, leukocytes, including DCs and T cells, and in fibroblastic cell lines. Finally, based on the information obtained in the study on CCR7 in DCs (147), we hypothesize that the independence between these two activities could be due to the fact that both functions could be governed by independent signaling modules.

Chemoattractant receptors are involved in multiple biological contexts both under homeostatic conditions and in multiple pathologies e.g. metastasis, and many chronic inflammatory diseases, including autoimmune diseases (5–8). Current models have provided a treasure trove of information on the mechanisms used by chemoattractant receptor-to control chemotaxis. The results obtained in this work can be useful to improve these models. Precise models showing the mechanisms used by these receptors to govern selectively directional sensing and chemokinesis can be useful to develop more effective treatments for the pathologies in which these receptors are involved.

## Data availability statement

The original contributions presented in the study are included in the article/supplementary material. Further inquiries can be directed to the corresponding author.

## Author contributions

JLR-F conceived and wrote the manuscript. OC-G performed important contributions to the manuscript, figures and Tables. All authors contributed to the article and approved the submitted version.

## Funding

This work was supported by grants SAF-2014-53151-R (Ministerio de Economía y Competitividad), SAF2017-83306-R (Ministerio de Ciencia, Innovación y Universidades), RIER (RETICS Program/Instituto de Salud Carlos III) (RD08/0075, RD16/0012/0007) and PID2020-114147RB-100 (Ministerio de Ciencia e Innovación).

## Acknowledgments

We acknowledge to all the present and past members of our laboratory for their important contribution to the study of chemokine receptors in dendritic cells.

## Conflict of interest

The authors declare that the research was conducted in the absence of any commercial or financial relationships that could be construed as a potential conflict of interest.

## Publisher’s note

All claims expressed in this article are solely those of the authors and do not necessarily represent those of their affiliated organizations, or those of the publisher, the editors and the reviewers. Any product that may be evaluated in this article, or claim that may be made by its manufacturer, is not guaranteed or endorsed by the publisher.
